# Blocking microglial activation of reactive astrocytes is neuroprotective in models of Alzheimer’s disease

**DOI:** 10.1186/s40478-021-01180-z

**Published:** 2021-04-26

**Authors:** Jong-Sung Park, Tae-In Kam, Saebom Lee, Hyejin Park, Yumin Oh, Seung-Hwan Kwon, Jae-Jin Song, Donghoon Kim, Hyunhee Kim, Aanishaa Jhaldiyal, Dong Hee Na, Kang Choon Lee, Eun Ji Park, Martin G. Pomper, Olga Pletnikova, Juan C. Troncoso, Han Seok Ko, Valina L. Dawson, Ted M. Dawson, Seulki Lee

**Affiliations:** 1 Russell H, Morgan Department of Radiology and Radiological Science, The Johns Hopkins University School of Medicine, Baltimore, MD 21205 USA; 2 Center for Nanomedicine at the Wilmer Eye Institute, The Johns Hopkins University School of Medicine, Baltimore, MD 21205 USA; 3 Neuroregeneration and Stem Cell Programs, Institute for Cell Engineering, The Johns Hopkins University School of Medicine, Baltimore, MD 21205 USA; 4 Department of Neurology, The Johns Hopkins University School of Medicine, Baltimore, MD 21205 USA; 5 Department of Physiology, The Johns Hopkins University School of Medicine, Baltimore, MD 21205 USA; 6 Solomon H. Snyder Department of Neuroscience, The Johns Hopkins University School of Medicine, Baltimore, MD 21205 USA; 7 Department of Pharmacology and Molecular Sciences, The Johns Hopkins University School of Medicine, Baltimore, MD 21205 USA; 8 College of Pharmacy, Chung-Ang University, Seoul, Republic of Korea; 9 D&D Pharmatech Inc., Bundang-gu, Seongnam-si, 13494 Republic of Korea; 10 Neuraly Inc., Gaithersburg, MD 20878 USA

**Keywords:** Alzheimer’s disease, GLP-1R agonist, GLP-1 receptor, Reactive astrocytes, NLY01, Microglia activation

## Abstract

Alzheimer’s disease (AD) is the most common cause of age-related dementia. Increasing evidence suggests that neuroinflammation mediated by microglia and astrocytes contributes to disease progression and severity in AD and other neurodegenerative disorders. During AD progression, resident microglia undergo proinflammatory activation, resulting in an increased capacity to convert resting astrocytes to reactive astrocytes. Therefore, microglia are a major therapeutic target for AD and blocking microglia-astrocyte activation could limit neurodegeneration in AD. Here we report that NLY01, an engineered exedin-4, glucagon-like peptide-1 receptor (GLP-1R) agonist, selectively blocks β-amyloid (Aβ)-induced activation of microglia through GLP-1R activation and inhibits the formation of reactive astrocytes as well as preserves neurons in AD models. In two transgenic AD mouse models (5xFAD and 3xTg-AD), repeated subcutaneous administration of NLY01 blocked microglia-mediated reactive astrocyte conversion and preserved neuronal viability, resulting in improved spatial learning and memory. Our study indicates that the GLP-1 pathway plays a critical role in microglia-reactive astrocyte associated neuroinflammation in AD and the effects of NLY01 are primarily mediated through a direct action on Aβ-induced GLP-1R^+^ microglia, contributing to the inhibition of astrocyte reactivity. These results show that targeting upregulated GLP-1R in microglia is a viable therapy for AD and other neurodegenerative disorders.

**Supplementary Information:**

The online version contains supplementary material available at 10.1186/s40478-021-01180-z.

## Introduction

Neurodegenerative disorders, including Alzheimer’s disease (AD), constitute a large unmet medical need as well as a massive and growing drain on the nation’s health care systems. Unlike other major disease areas, therapeutic drug development for AD remains challenging and the small number of approved drugs only mitigate the symptoms of the disease without addressing its underlying causes [1, 22]. The pathogenesis of AD is complex but involves pathologic beta-amyloid (Aβ) and tau protein accumulation in plaques and tangles, respectively, in brain tissue [18, 35]. Multiple experimental therapies targeted towards reducing Aβ accumulation have demonstrated promise in animal models, however, they have failed in clinical trials in AD patients so far. There are many additional drugs in clinical investigation, the majority of which are anti-amyloid or anti-tau agents, enzyme inhibitors, neurotransmitter based or anti-inflammatory agents [7, 11]. However, the FDA has not approved a new drug for AD since 2003 [11].

The involvement of neuroinflammation manifested by microglia and astrocytes in AD is supported by a wealth of clinical and molecular evidence [14, 15, 32, 33]. Microglia are the resident macrophages of the central nervous system (CNS) and become activated disease-associated microglia (DAM) in response to systemic inflammation or neurodegeneration, [31, 33, 34]. Chronic activation of microglia can potentially cause neurotoxicity and facilitate neurodegenerative disease progression. Recently, it was shown that activation of microglia leads to the conversion of resting astrocytes to reactive astrocytes via secretion of IL-1α, TNFα, and C1q in a variety of neurodegenerative disorders including AD and Parkinson’s disease (PD) [23, 24, 38]. Therefore, a therapeutic agent that could inhibit microglia and astrocyte activation without off-target toxicity could potentially be developed as a universal neuroprotective drug for neurodegenerative disorders, including AD.

Glucagon-like peptide-1 (GLP-1) is a gut-derived incretin, secreted primary from L cells of the intestine in response to meal intake [17]. The GLP-1 receptor (GLP-1R) is expressed in pancreatic islets, brain, heart, and the gastrointestinal tract. GLP-1 lowers blood sugar levels in a glucose-dependent manner by binding the GLP-1R on pancreatic beta cells, resulting in the enhanced secretion of insulin. This role of GLP-1R in controlling blood sugar levels has resulted in the approval of GLP-1R agonists for treatment of type 2 diabetes [9]. Besides its role in glucose homeostasis, GLP-1 is involved in a variety of cellular functions including apoptosis and cell viability and plays a role as a neuropeptide that regulates many autonomic and neuroendocrine functions [16, 28]. Recently, we reported in models of PD that pathologic α-synuclein induces microglia activation and increases microglia GLP-1R expression in vitro and in vivo [38]. NLY01 is a pegylated exendin-4, a CNS penetrating and long-acting GLP-1R agonist with extended half-life in non-human primates [38]. We discovered that NLY01 blocks pathologic α-synuclein-induced microglial activation in vitro and in vivo as well as, inhibits induction of inflammatory mediator, IL-1α, TNFα, and C1q, preventing astrocyte reactivity. In two PD mouse models associated with α-synuclein pathology, subcutaneously (s.c.) injected NLY01 showed strong anti-PD efficacy by blockage of microglial activation and reactive astrocyte conversion leading to synergistic anti-inflammatory and neuroprotective effects as well as improvement in cognition and neurobehavior without any adverse effects.

Neuroinflammation associated with microglia and astrocyte interaction as well as increased reactive astrocytes population in PD is also seen in AD [5, 12, 19, 20, 24, 33]. However, the biological pathways that are associated with GLP-1R signaling in glial cells and neuroinflammation as well as neurodegeneration in AD are not fully understood. We hypothesized that NLY01 could reduce or ameliorate the AD phenotype by inhibiting microglia-mediated conversion of reactive astrocytes through GLP-1R^+^ microglia during the progression of AD pathology. Herein, we explored the mechanisms of GLP-1R sensitization in the Aβ-associated microglia-astrocyte-neuron triad and tested the efficacy of NLY01 in two AD models. In vivo, NLY01 is profoundly neuroprotective against the 5xFAD and 3xTg-AD mouse models and restores memory functions via direct actions on microglial-mediated conversion of resting astrocytes to reactive astrocytes. We demonstrated that NLY01 selectively inhibits Aβ-induced microglial activation and blocks reactive astrocyte conversion, sparing neurons in AD as seen in PD models. These results warrant further investigation into microglia-targeted GLP-1R agonists as anti-AD therapeutic strategies.

## Materials and methods

### AD patients

The written informed consent approved by the Johns Hopkins Institutional Review Boards (Approval No. NA00032761) was provided to and signed by all study subjects. Human post-mortem brain tissues were obtained from the brain donation program of the Alzheimer’s disease research center at Johns Hopkins Medical Institutions (JHMI) in compliance with local Institutional Review Board and HIPAA (Health Insurance Portability and Accountability Act) regulations. Detailed patient information is summarized in Additional file 1: Table S1 (online resource).

### Mice

All animal experiments were approved by the Johns Hopkins Medical Institute Animal Care and Use Committee and performed according to the Guide to the Care and Use of Animals laboratory animal manual of the National Institute of Health. B6129SF2 (WT), 5xFAD (B6SJL-Tg (APPSwFlLon, PSEN1*M146L*L286V)6799Vas/Mmjax), and 3xTg-AD (B6;129-Psen1^tm1Mpm^Tg(APPSwe,tauP301L)1Lfa/Mmjax) mice [30] were purchased from the Jackson Laboratory (Jackson Laboratories, Bar Harbor, ME). In the 5xFAD mice study, 3-month-old mice were administered PBS or NLY01 (1 or 10 mg/kg) by subcutaneous injection twice a week for 4 months. In the 3xTg-AD mice study, 7-month-old male mice were treated with PBS or NLY01 (1 or 10 mg/kg) by subcutaneous injection twice a week for 5 months. PBS treated groups served as control.

### Morris water maze

The circular pool (120 cm in diameter and 35 cm in height) was filled with water and a nontoxic water-soluble white dye. The pool was divided into four quadrants of equal area. A platform (8 cm in diameter) was placed in the center of one of the quadrants and 1 cm below the water surface. Various prominent visual cues were contained in the pool as spatial references. The day before the experiment was dedicated to swimming training for 60 s in the absence of the platform. The mice were then given three trials each day for five consecutive days, with an inter-trial interval of 15 min, and the escape latencies were recorded. This parameter was averaged over trials for each day and for each mouse. Once the mouse located the platform, it remained on it for at least 10 s. If the mouse did not find the platform within 60 s, it was placed on the platform for 10 s by the experimenter. On day 6, the platform was removed from the pool for a probe trial to test reference memory. The probe trial was performed with a 60 s cut-off time. The time spent in target quadrant and swimming speed were recorded. A video tracking system (ANY-maze system, Wood Dale, IL, USA) was used to record data.

### Passive avoidance test

For training, each mouse was placed in a lighted compartment box. When the mouse crossed over to the dark compartment, it received a mild electrical shock (0.25 mA/1 s). This initial latency to enter the dark compartment was recorded as the baseline measure. After 24 h, each mouse was again placed in the light compartment, and the latency to return to the dark compartment was measured as an index of passive avoidance.

### Y-maze

Each mouse was allowed to freely explore the three arms of the maze (Y shape; 40 × 8 × 15 cms) for 8 min. The number of arm entries and the number of triads, sequence of three consecutive arm entries, were recorded in order to calculate the percentage of alternation. An entry occurred when all four limbs are within the arm.

### Exendin-4 ELISA

For peptide extraction from tissues, brain tissues were homogenized in 1% trifluoroacetic acid (Sigma-Aldrich, ST. Louis, MO, USA) and heated for 10 min at 100 ℃. The tissue homogenates were centrifugated at 12,000 rpm for 30 min at 4 ℃ and then the supernatant was extracted in a SEP-COLUMN containing 200 mg of C18 (Phoenix Pharmaceuticals, Burlingame, CA, USA). The extracted peptide was dried in the lyophilizer and measured using an Exendin-4 EIA Kit (Phoenix Pharmaceuticals), according to the manufacturer’s protocol.

### Glucose level measurement

Three month old 5 × FAD mice were administered 2 mg/kg NLY01 by intravenous injection daily for 5 days. On day 5, three hours after the last injection, blood glucose levels were measured in WT and 5 × AD mice. 5 × FAD mice who were treated with NLY01 for 4 months were used to measure brain glucose levels. Levels of blood and brain glucose were measured using a Glucose assay kit (Abcam, Cambridge, MA, USA) according to the manufacturer’s protocol.

### Quantitative PCR

Total RNAs from hippocampal tissues and primary cells were extracted using a RNeasy mini kit (Qiagen Sciences, Inc., Germantown, MD, USA) and quantified using a UV–Vis spectrophotometer (NanoDrop 2000, Thermo Fisher Scientific Inc., Wilmington, DE, USA). RNA was reverse-transcribed using a high capacity cDNA reverse transcription kit (Applied Biosystems, Carlsbad, CA, USA). cDNAs were amplified with PowerUp™ SYBR Green Master Mix (Applied Biosystems) on a StepOnePlus™ system (Applied Biosystems). The amounts of cDNA within each sample were normalized to 18sRNA or glyceraldehyde 3-phosphate dehydrogenase (GAPDH), and relative mRNA levels were analyzed using the 2^−ΔΔct^ method. Additional file 1: Table S2 (online resource) shows the primer sequences.

### Immunostaining

Brain tissues were fixed by perfusion with 4% paraformaldehyde (PFA), transferred to a 30% sucrose solution, and prepared in to 40 μm sections using a freezing microtome. The sections and neurons were blocked with BlockAid™ blocking solution (Invitrogen, Carlsbad, CA, USA) for 1 h and then immunostained using GLP-1R (Santa Cruz Biotechnology, Inc., Santa Cruz, CA, USA), Iba-1 (Abcam), GFAP (Cell signaling Technology, Danvers, MA, USA), MBP (Invitrogen), TBR1 (Abcam), CTIP2 (Abcam), SATB2 (Abcam), MAP2 (Cell signaling Technology, Millipore), and 4G8 (BioLegend, San Diego, CA, USA) antibodies at 4 ℃ overnight. The sections were then incubated with Alexa Flour 488 (Invitrogen) or Alexa Flour 594 (Invitrogen) conjugated secondary antibodies for 1 h at room temperature (RT). DAPI (Vector laboratories, Burlingame, CA, USA) was used for nucleus staining. The analysis was performed using a Zeiss LSM710 confocal microscope (Carl Zeiss, Göttingen, Germany). For immunohistochemistry, we used a Vectastain ABC IHC kit (Vector Laboratories, Burlingame, CA, USA) and Nikon Eclipse E600 microscope with a DS-Fi2 digital camera (Nikon, Melville, NY, USA).

### Western blot

Cells lysed by RIPA buffer (Thermo Fisher Scientific Inc.) and hippocampal tissues from mice were homogenized in T-per buffer (Thermo Fisher Scientific Inc.) and centrifuged at 12,000 rpm for 10 min at 4 ℃. In each sample, 10 μg of protein was separated with SDS/PAGE followed by transfer to the PVDF membranes (Bio-Rad, California, WC, USA). The membranes were washed in TBS with 0.001% Tween 20 and blocked in 3% BSA for 1 h at RT. Membranes and primary antibodies of GLP-1R (Santacruz), GFAP (Cell signaling Technology), Iba-1 (Abcam), Tuj1 (Biolegend, San Diego, CA, USA), C3 (Abcam), MAP2 (Cell signaling Technology), BDNF (Abcam), Bcl-2 (Cell signaling Technology), PSD95 (Cell signaling Technology), β-actin (Santacruz), and GAPDH (Santacruz) were incubated overnight at 4 ℃. The antibodies used in this study are summarized in Additional file 1: Table S3 (online resource). Membranes were then incubated with HRP-conjugated secondary antibodies for 1 h at RT and visualized with ECL Western Blotting Substrate (Promega Corporation, Madison, WI, USA). ImageJ software (National institutes of Health) was used for protein quantification.

### Preparation of oligomeric Aβ_1-42_

Oligomeric Aβ_1-42_ were generated as previously described [21]. Hexafluoroisopropanol (HFIP)-treated synthetic Aβ_1-42_ peptides (rPeptide, Bogart, GA, USA) were dissolved in dimethyl sulfoxide (DMSO) and further diluted in phosphate-buffered saline (PBS) to obtain a 250 μM stock solution. The stock solution was incubated at 4 ℃ for at least 24 h and stored at − 80 ℃ until use. Before use, the solution was centrifuged at 12,000 g for 10 min and the supernatant was used as an oligomeric Aβ (ADDL). The oligomeric status of Aβ_1-42_ was evaluated by Western blot analysis. 1 μM of oligomeric Aβ_1-42_ was used for cellular treatment.

### Preparation of primary microglia, astrocytes, and neurons

Primary microglia and astrocytes were obtained from the brains of postnatal mouse pups (P1). The extracted brains, after removal of the meninges, were washed in DMEM/F12 (Gibco, Gaithersburg, MD, USA) supplemented with 10% heat-inactivated FBS, 50 U/mL penicillin, 50 μg/mL streptomycin, 2 mM L-glutamine, 100 μM non-essential amino acids, and 2 mM sodium pyruvate (DMEM-F12 complete medium) three times. A single cell suspension was obtained via trituration of 0.25% trypsin–EDTA treated brains and removal of cell debris and aggregates with a 100 μm nylon mesh. The final single cell suspension was cultured in T-175 flasks for 13 days, with a complete medium change on day 6. The mixed glial cell cultures were separated into a magnetic-bound microglia enriched fraction and pour-off astrocyte enriched fractions using the EasySep Mouse CD11b Positive Selection Kit (StemCell, Cambridge, MA, USA) according to the manufacturer instructions. The microglia conditioned medium (MCM), collected from the primary microglia treated with oligomeric Aβ (Aβ MCM) with either PBS or NLY01 treatment, was applied to primary astrocytes for 24 h. The astrocyte conditioned medium (ACM) was collected using a complete, Mini, EDTA-free Protease Inhibitor Cocktail (Sigma-Aldrich) and concentrated with an Amicon Ultra-15 centrifugal filter unit (10 kDa cutoff) (Millipore, Burlington, MA USA) until it was approximately 50-X concentrated. To determine neuronal cell death, total protein concentration was quantified using a Pierce BCA protein assay kit (Thermo Scientific), and 15 or 50 μg/mL of total protein was applied to mouse primary cortical neurons or human cortical neurons, respectively. Adult microglia were obtained from 8-month-old C57BL/6 mice as previously described [27]. The sgRNAs targeting GLP-1R (KO1, 5′-CCAGGAGTGGCGCTTCCGTG-3′) were subcloned into a pLentiCRISPR vector (Addgene, Cambridge, MA, USA) and the lentivirus was generated as previously described [3]. GLP-1R levels were assessed by immunoblot from transduced primary microglia.

Primary cortical neurons were cultured from embryonic day 15.5 pups of CD1 mice (Jackson Laboratories). The neurons were plated on poly-L-lysine coated plates and maintained in neurobasal medium (Gibco) containing B-27, 0.5 mM L-glutamine, penicillin and streptomycin (Invitrogen). Half of the medium was exchanged every 3 days. Mouse primary neurons are Tuj1-positive but GFAP- and Iba-1-negative as assessed by western blotting.

### Preparation of human cortical neurons

Human embryonic stem cell (hES) derived cortical neurons were prepared using the RONA protocol previously described [37]. H1 Human embryonic stem cells were cultured on inactivated mouse embryonic fibroblasts supplemented with human ESC medium containing Dulbecco’s modified Eagle’s medium/nutrient mixture F-12 (DMEM/F12; Invitrogen), 20% knockout serum replacement (Invitrogen), fibroblast growth factor 2 (bFGF; 10 ng/mL; Thermo Fisher), 1 mM GlutaMAX (Invitrogen), 100 μM nonessential amino acids (Invitrogen), and 100 μM 2-mercaptoethanol (Invitrogen). The hES cells were harvested using collagenase and were cultured in suspension for 7 days in hES cell media without bFGF to create embryonic bodies (EB). Thereafter, the EB were transferred to matrigel coated plates to allow complete attachment and supplemented with N2 induction medium containing DMEM/F12 (Invitrogen), 1% N2 supplement (Invitrogen), 100 μM MEM nonessential amino acid solution (Invitrogen), 1 mM GlutaMAX (Invitrogen), and heparin (2 μg/mL; Sigma-Aldrich). When the EB created neural aggregates (also called RONAs), we selected out the neural progenitors under a dissection microscope. The aggregates were collected in suspension for a day as neurospheres, dissociated into single cells, and plated on poly-D-lysine/laminin coated plate to induce further differentiation. The progenitor cells were supplemented with Neurobasal media, B27 (Invitrogen), ascorbic acid (0.2 mM; Sigma), dibutyryl adenosine 3′,5′-monophosphate (cAMP; 0.5 mM; Sigma), brain-derived neurotrophic factor (BDNF; 20 ng/mL; PeproTech, Rocky Hill, NJ, USA), and glial cell line–derived neurotrophic factor (GDNF; 20 ng/mL; PeproTech), until they were fully matured as cortical neurons. Human cortical neurons are composed of Tuj1–positive neuronal cells (> 90%) with about 5–10% GFAP–positive astrocytes [37].

### Cytokine analysis

The conditioned medium from primary microglia 18 h after oligomeric Aβ with either PBS or NLY01 treatment were used for detection of secreted cytokines TNFα, IL-1α, IL-1β (Thermo Fisher Scientific), and C1q (LSBio, Seattle, WA, USA) by mouse ELISA kits.

### Cell death and viability analysis

Cell death and viability were tested using propidium iodide staining and Alamar Blue assays, respectively, according to the manufacturer’s protocol. Primary cultured cortical neurons were treated with ACM derived from astrocytes treated either alone or together with oligomeric Aβ and NLY01 for 24 h. Cell death was determined by unbiased objective computer-assisted cell counting after staining of all nuclei with 7 μM Hoechst 33,342 (Invitrogen) and dead cell nuclei with 2 μM propidium iodide (Invitrogen). The numbers of total and dead cells were counted with the Axiovision 4.3 software (Carl Zeiss).

### Statistical analysis

Data were analyzed using GraphPad Prism 7 (GraphPad Software, San Diego, CA, USA). All data were expressed as mean $\pm$ SEM. Student’s t-test (two-tailed) was used for single comparison. One-way ANOVA analysis of variance with Dunnett’s multiple comparisons test was used for multiple group analysis. A value of *p* < 0.05 was considered statistically significant.

## Results

### NLY01 ameliorates cognitive deficits in 5 × FAD mice

**Fig. 1:**
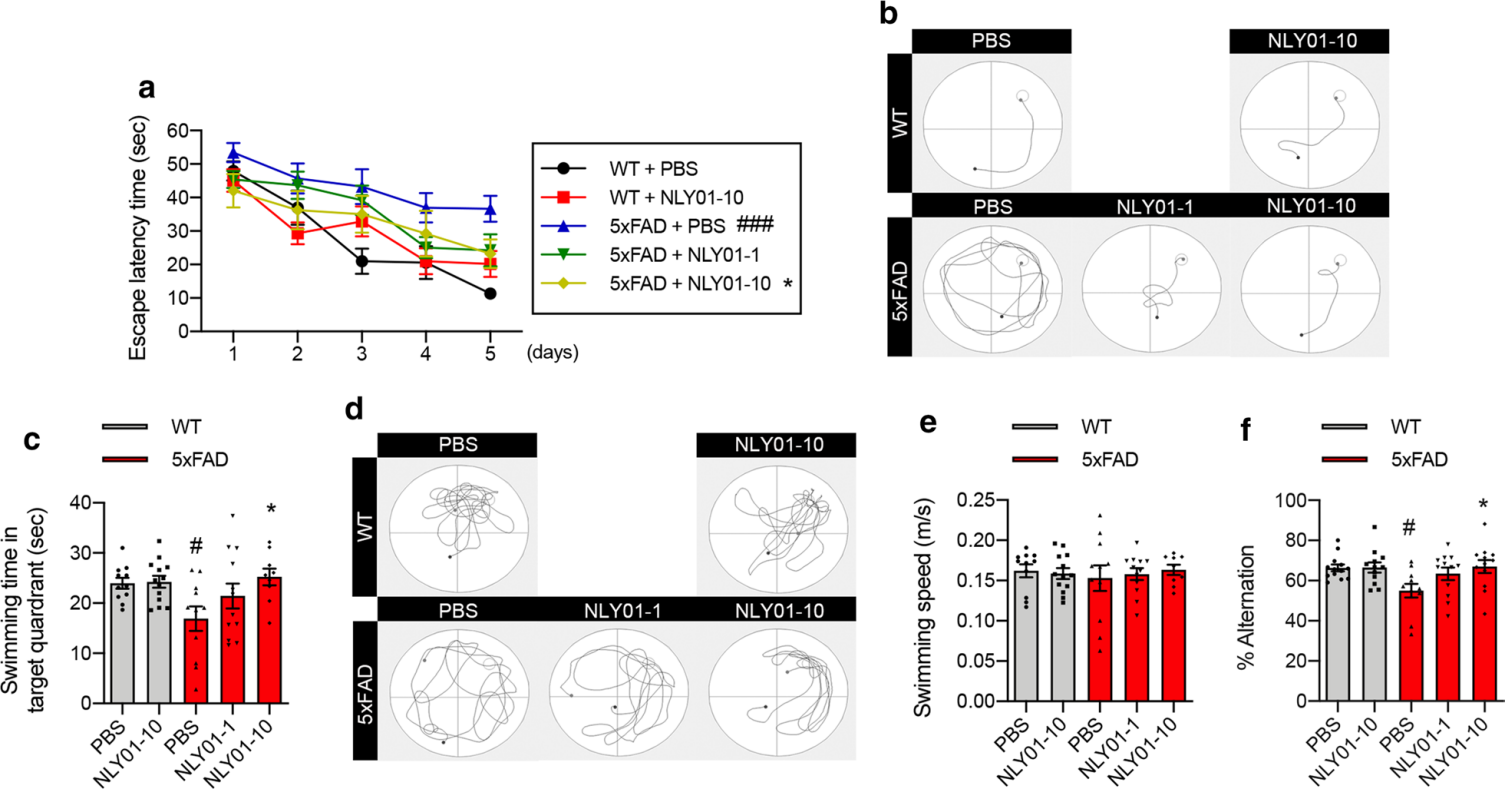
NLY01 ameliorates behavioral deficits in 5xFAD mice. 3-month-old 5xFAD mice were treated with PBS or NLY01 (1 or 10 mg/kg) subcutaneously (s.c.) for 4 months (*n* = 9–13 per group). **a** Mice were trained and tested on the spatial memory version of the Morris water maze (MWM; indicated by solid lines at 60 s escape latency) (*n* = 9–13 per group). **b** Representative swimming traces for training trials on day 5 are shown. **c** Mice were given a memory probe with the platform removed at 24 h after the last training trial (*n* = 9–13 per group). **d** Representative swimming traces of the memory test at 24 h after the last training trial. **e** Swimming speed of WT mice and 5xFAD mice treated with vehicle or NLY01 (1 or 10 mg/kg) (*n* = 9–13 per group). **f** Mice were tested on a spatial alternation task in a Y-maze (*n* = 11–13 per group). Data are shown as the mean $\pm$ SEM. *p* values were determined by one-way ANOVA. ^#^*p* < 0.05, ^###^*p* < 0.001 versus WT + PBS; *p < 0.05 versus 5xFAD + PBS

To determine the effects of NLY01 in an AD mouse model, 3-month-old 5xFAD mice were treated with either PBS or NLY01 (1 or 10 mg/kg), twice weekly via s.c. injection, for a period of 4 months. These mice contain five familial AD mutations (APP_swe_, APP_I716V_, APP_V717I_, Psen_1M146L_, and Psen_1L286V_) and accumulate high levels of amyloid deposition within 2 months of age with gliosis, resulting in impaired memory function [29]. In the Morris water maze (MWM) test, we observed a marked decline in performance in PBS treated 5xFAD mice compared to WT mice (Fig. 1a, b). In contrast, NLY01 treated 5xFAD mice showed improved spatial learning across the five day training period (Fig. 1a, b). Probe trials were conducted 24 h after the last training trial with the removed platform. 5xFAD mice treated with NLY01 demonstrated significantly increased time in the target quadrant in the probe trial compared to that of non-treated 5xFAD mice (Fig. 1c, d). No difference in swimming speed was observed between all groups (Fig. 1e). We further tested memory function using the Y-maze task. NLY01 treated 5xFAD mice performed similarly to WT, with increased spontaneous alternation compared to non-treated 5xFAD (Fig. 1f). NLY01 did not alter the blood and the brain glucose levels as well as body weight in the WT and 5xFAD mice (Additional file 1: Fig. S1, online resource).

### GLP-1R levels are upregulated in AD and microglia stimulated with oligomeric Aβ_1–42_

**Fig. 2:**
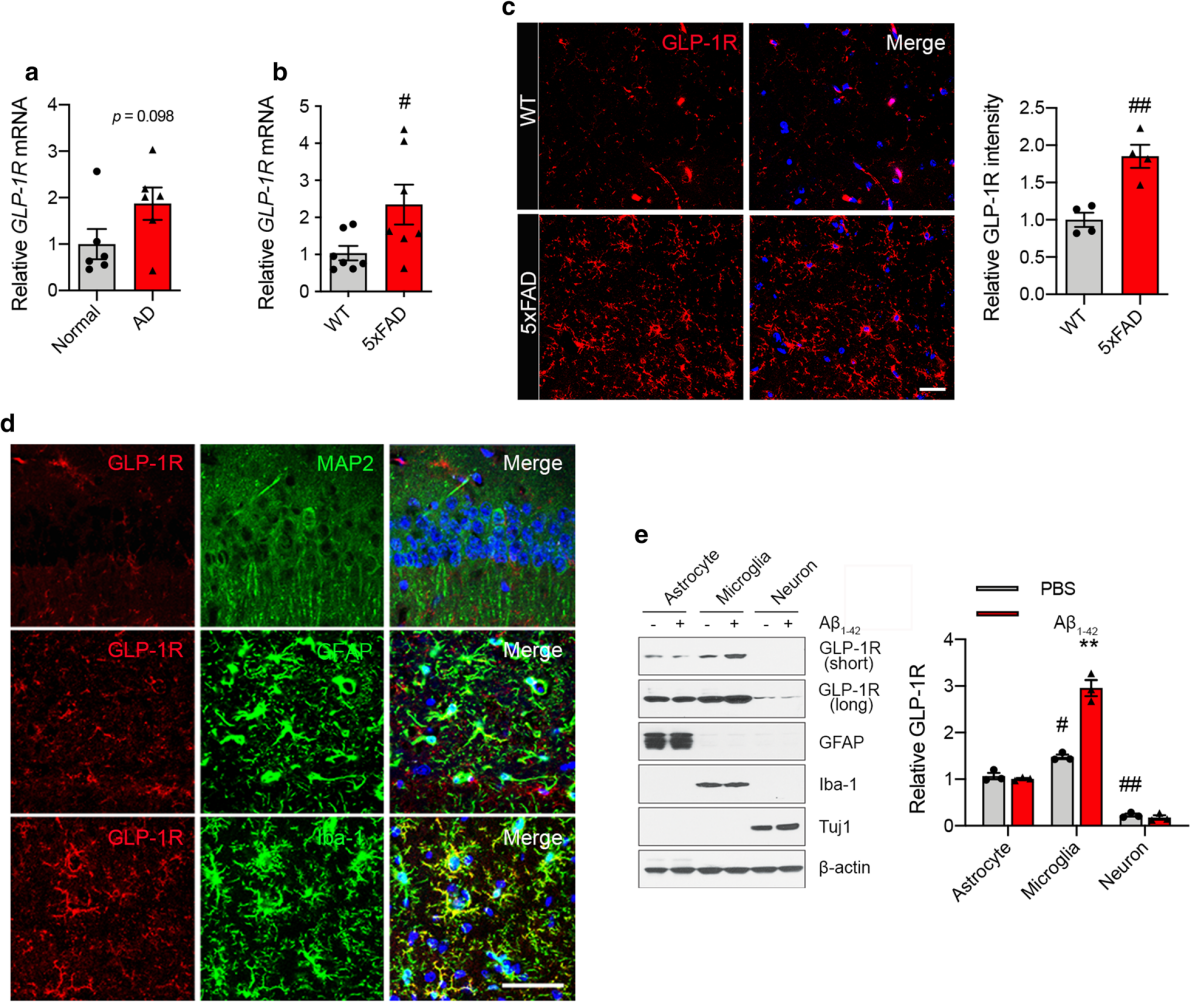
GLP-1R is increased in the hippocampus of AD brains and in microglia exposed to Aβ_1-42_. **a**, **b** Relative *GLP-1R* mRNA expression in the hippocampus from the brain of **a** AD patients (*n* = 6 per group) and **b** 5xFAD mice (7-month-old; *n* = 7 per group). **c** Representative confocal images with GLP-1R (red) and DAPI (blue) in the hippocampus of 5xFAD mice (scale bars, 20 μm) and quantification of the GLP-1R immunostaining (*n* = 4 per group). **d** Representative confocal images of immunostaining with GLP-1R (red), MAP2 (green; upper), GFAP (green; middle), Iba-1 (green; lower), and DAPI (blue) in the hippocampus from 3xTg-AD mice (12-month-old; *n* = 3) (scale bar, 50 μm). **e** Primary astrocytes, microglia, and neurons were incubated with oligomeric Aβ_1-42_ (1 μM) for 4 h. The levels of GLP-1R (exposed to a short or long time), GFAP, Iba-1, Tuj1, and β-actin were determined using Western blot. Quantification of GLP-1R is shown as relative protein expression normalized to β-actin (*n* = 3 biologically independent cell cultures). Data are shown as the mean $\pm$ SEM. *p* values were determined by two-tailed unpaired t-test or one-way ANOVA. ^#^*p* < 0.05, ^##^*p* < 0.01 versus WT or astrocyte + PBS; **p < 0.01 versus microglia + PBS

The levels of GLP-1R expression in human postmortem hippocampal tissues from age-matched control and AD patients was assessed by quantitative PCR (qPCR). We observed an increase in mRNA expression of *GLP-1R* in AD patients’ hippocampal tissues compared with control (Fig. 2a). Similarly, the hippocampal expression of mRNA and protein for GLP-1R in 5 × FAD mice (7 months) was significantly elevated compared to WT, as determined by GLP-1R immunoreactivity and Western blot (Fig. 2b, c; Additional file 1: Fig. S2a, online resource). Consistent with 5xFAD mice, we observed upregulated GLP-1R expression in the hippocampus from 3xTg-AD mice (Additional file 1: Fig. S2b, c, online resource). Increased GLP-1R immunoreactivity was mainly localized to ionized calcium-binding adapter molecule (Iba-1)^+^ microglia (Fig. 2d). GLP-1R immunoreactivity showed limited colocalization with glial fibrillary acidic protein (GFAP)^+^ astrocytes and did not colocalize with microtubule-associated protein 2 (MAP2)^+^ neurons (Fig. 2d**)**. Further, we observed that GLP-1R immunoreactivity showed limited colocalization with myelin basic protein (MBP)^+^ oligodendrocytes (Additional file 1: Fig. S2d, online resource). These results were confirmed in cultured microglia with oligomeric Aβ_1–42_ stimulation. In vitro, GLP-1R protein is highly expressed in primary mouse-derived microglia and astrocytes compared to neurons. When glial and neurons were treated with oligomeric Aβ_1-42_, GLP-1R protein levels were upregulated in microglia (Fig. 2e). Since GLP-1R is upregulated in AD and models of AD microglia, this is the likely site of action of GLP-1R.

### NLY01 attenuates Aβ_1–42_ induced microglia activation through GLP-1R

**Fig. 3:**
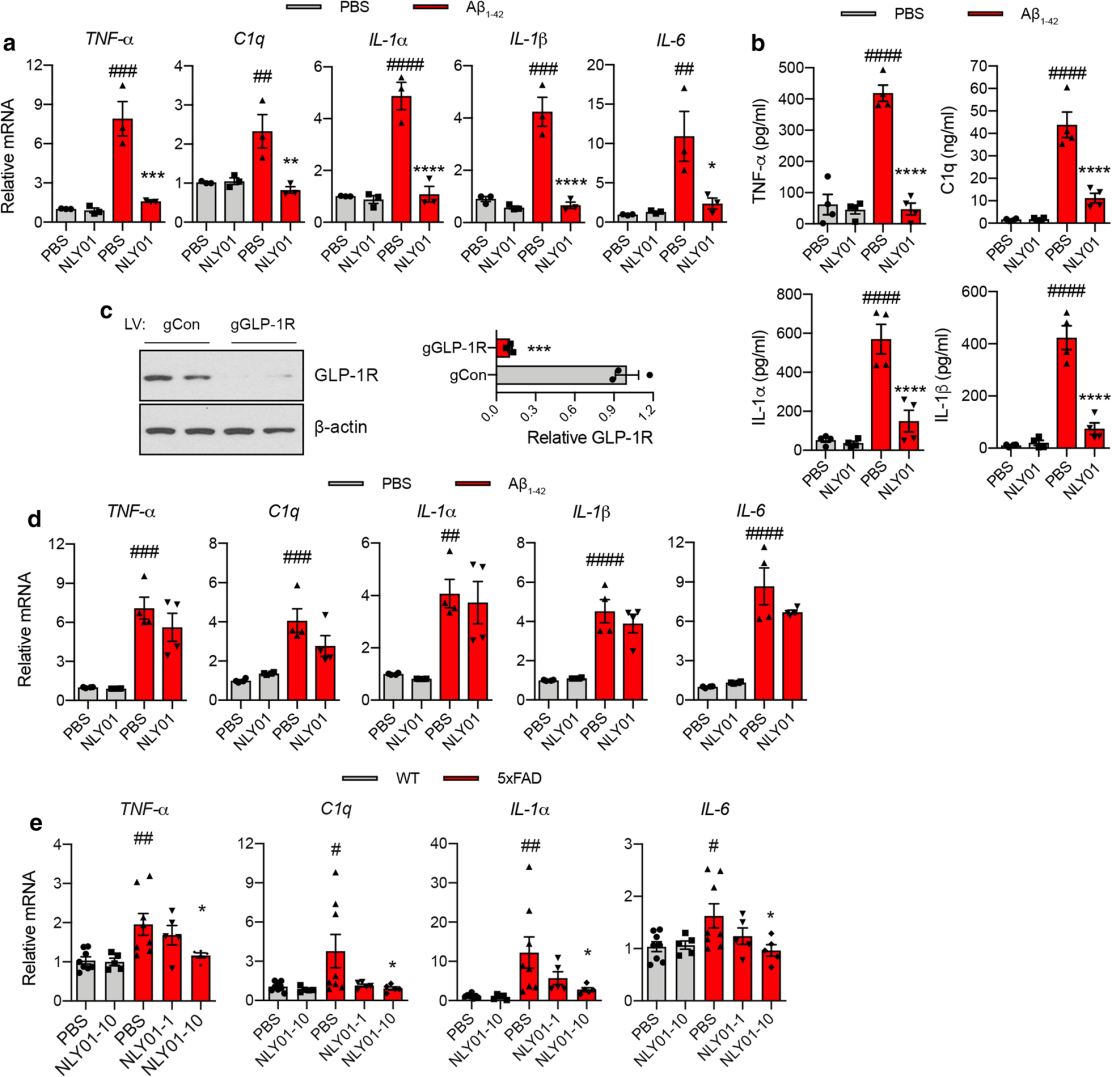
NLY01 suppresses Aβ-induced microglia activation. **a** Primary microglia were pre-treated with PBS or NLY01 (1 μM) for 30 min, and then further incubated with oligomeric Aβ_1-42_ (1 μM) for 4 h. mRNA levels of *TNF-α*, *C1q*, *IL-1α*, *IL-1β*, and *IL-6* were determined using qPCR (*n* = 3 biologically independent cell cultures). **b** Cytokine release in media from primary microglia incubated with oligomeric Aβ_1-42_ (1 μM) for 18 h. TNF-α, C1q, IL-1α, and IL-1β were measured by ELISA (*n* = 4 biologically independent cell cultures). **c** GLP-1R and β-actin expression in lenti-CRISPR/Cas9 mediated GLP-1R KO microglia. Quantification of GLP-1R shown as relative protein expression normalized to β-actin (*n* = 3 biologically independent cell cultures). **d** mRNA levels of *TNF-α*, *C1q*, *IL-1α*, *IL-1β*, and *IL-6* in oligomeric Aβ_1-42_ (1 μM) treated lenti-CRISPR/Cas9 mediated GLP-1R KO microglia (*n* = 4 biologically independent cell cultures). **e** Levels of *TNF-α*, *C1q*, *IL-1α*, and *IL-6* analyzed by qPCR in the hippocampus of 5xFAD mice treated with PBS or NLY01 (*n* = 5–8 per group). Data are shown as the mean $\pm$ SEM. *p* values were determined by two-tailed unpaired t-test or one-way ANOVA. ^#^*p* < 0.05, ^##^*p* < 0.01, ^###^*p* < 0.001, ^####^*p* < 0.0001 versus Control + PBS or WT + PBS; *p < 0.05, **p < 0.01, ***p < 0.001, ****p < 0.0001 versus Aβ_1-42_ + PBS or 5xFAD + PBS

When primary microglia were treated with oligomeric Aβ_1–42_ for 4 h, Aβ_1–42_ significantly induced mRNA expression of *TNF-α, C1q, IL-1α, IL-1β*, and *IL-6* as determined by qPCR (Fig. 3a). ELISA results showed protein levels for TFN-α, C1q, IL-1α and IL-1β were also increased in microglia activated by oligomeric Aβ_1–42_ (Fig. 3b). When microglia were pre-treated with NLY01 for 30 min, NLY01 strongly suppressed production of cytokines induced by oligomeric Aβ_1–42_ both at the mRNA and protein levels (Fig. 3a, b). To ascertain whether NLY01 attenuated Aβ_1–42_-induced microglia activation via upregulated GLP-1R, GLP-1R KO microglia were generated by CRISPR/Cas9 (Fig. 3c) and were treated with oligomeric Aβ_1–42_ with or without NLY01. Oligomeric Aβ_1–42_ strongly induced mRNA expression of *TNF-α, C1q, IL-1α, IL-1β* and *IL-6* in GLP-1R KO microglia, which was not blocked by NLY01 treatment (Fig. 3d). Thus, our data suggest that the inhibitory effect of NLY01 on Aβ_1–42_-induced microglia activation is primary through microglial GLP-1R. As observed in our primary microglia results, 5xFAD mice demonstrated increased mRNA expression of *TNF-α, C1q, IL-1α,* and *IL-6* in hippocampus and this induction was strongly blocked by NLY01 treatment (Fig. 3e). In addition, we observed the protective role of NLY01 in primary microglia isolated from adult WT mouse hippocampus treated with Aβ_1–42_ (Additional file 1: Fig. S3, online resource).

### NLY01 prevents reactive astrocyte conversion induced by Aβ_1-42_ activated microglia

**Fig. 4:**
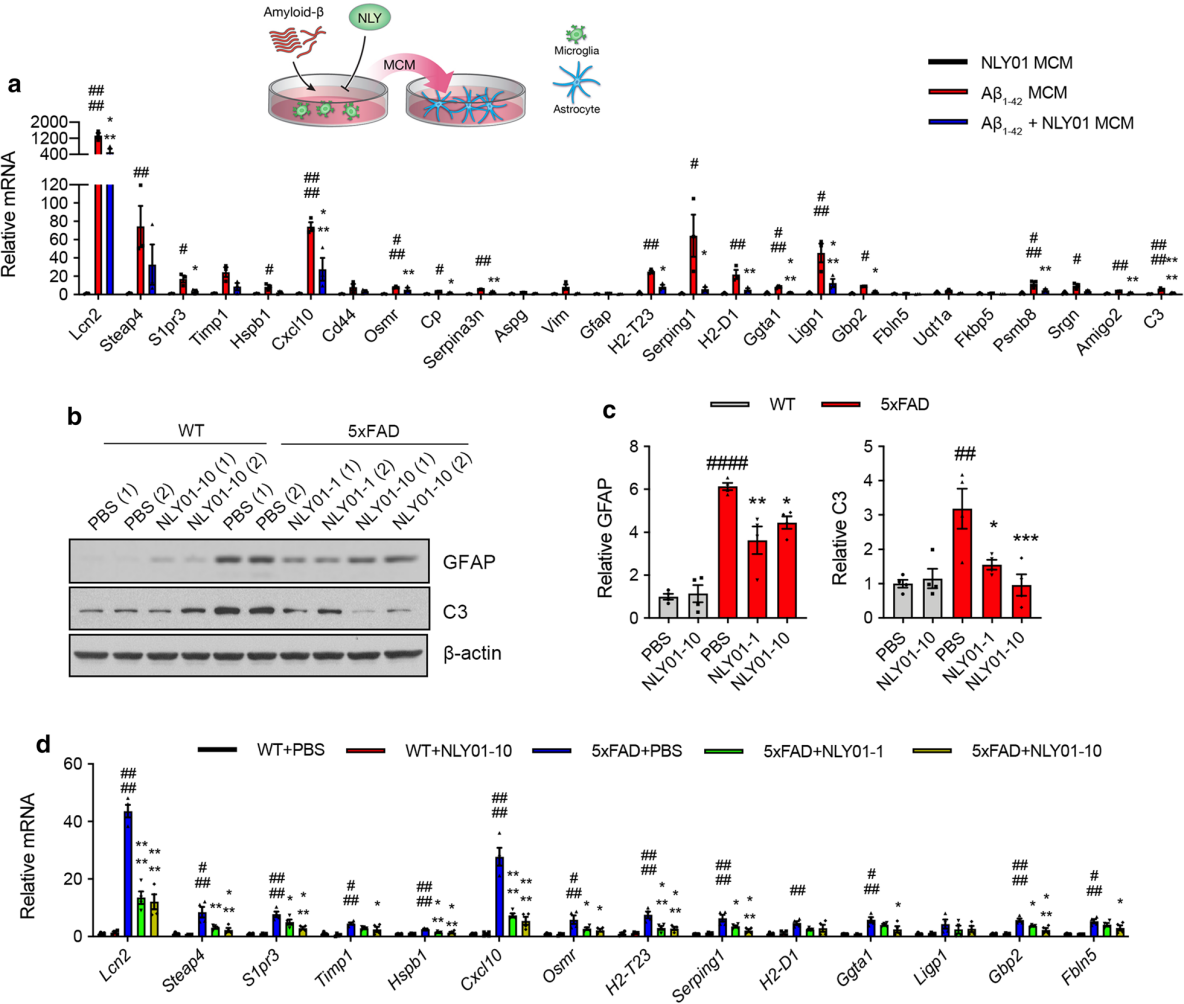
Inhibition of Aβ-induced reactive astrocytes by NLY01. **a** Schematic diagram showing the treatment of astrocytes with MCM from Aβ_1-42_ treated microglia with or without NLY01. mRNA levels of primary astrocytes 24 h post treatment with MCM from oligomeric Aβ_1-42_ (1 μM)-activated microglia with or without NLY01 (1 μM) is shown (*n* = 3 biologically independent cell cultures). **b** Levels of GFAP, C3, and β-actin in the hippocampus of WT and 5 × FAD mice treated with PBS or NLY01 using Western blot. **c** Relative GFAP and C3 protein levels were normalized versus β-actin levels (*n* = 4 per group). **d** mRNA levels of astrocyte signatures in the isolated astrocytes from the hippocampus of WT and 5 × FAD mice treated with PBS or NLY01 using qPCR. GAPDH was used to normalize for the amounts of cDNA (*n* = 4 per group). Data are shown as the mean $\pm$ SEM. *p* values were determined by one-way ANOVA. ^#^*p* < 0.05, ^##^*p* < 0.01, ^###^*p* < 0.001, ^####^*p* < 0.0001 versus Control MCM or WT + PBS; *p < 0.05, **p < 0.01, ***p < 0.001, ****p < 0.0001 versus Aβ_1-42_ MCM or 5xFAD + PBS

Since NLY01 efficiently blocks microglia activation stimulated by oligomeric Aβ_1–42_, we hypothesized that NLY01 prevents the formation of reactive astrocytes. To determine if Aβ_1–42_-induced microglia facilitate reactive astrocyte formation, oligomeric Aβ_1–42_ (1 μM) microglial conditioned media (MCM) was applied to primary mouse astrocytes for 24 h and mRNA levels of astrocyte-associated genes were assessed by qPCR. Aβ_1–42_ MCM induced mRNA expression of *Lcn2, Osmr, Serpina3n, Ggta1,* and *C3*—genes known to be associated with reactive astrocytes [10, 12, 24] (Fig. 4a; Additional file 1: Fig. S4, Table S4, online resource). In contrast, NLY01 treated MCM prevented the induction of reactive astrocyte-associated genes (Fig. 4a; Additional file 1: Table S4, online resource). To determine whether prevention of microglia activation by NLY01 results in suppression of reactive astrocyte conversion in vivo, we characterized the population of astrocytes in vehicle and NLY01 treated mice. 5xFAD mice demonstrated strong GFAP^+^ immunoreactivity in the hippocampus compared to WT mice, which was blocked by NLY01 (Additional file 1: Fig. S5a, online resource). Western blot analysis confirmed increased levels of GFAP and reactive astrocytes marker C3 in the hippocampus and that these levels were blocked by NLY01 (Fig. 4b, c). Importantly, NLY01 significantly prevented induction of reactive astrocyte signature genes, *Lcn2, Osmr,* and *Ggta1* in the 5xFAD mice (Fig. 4d; Additional file 1: Fig. S5b, Table S5, online resource).

### NLY01 rescues neuronal cell death by prevention of reactive astrocyte conversion

**Fig.5:**
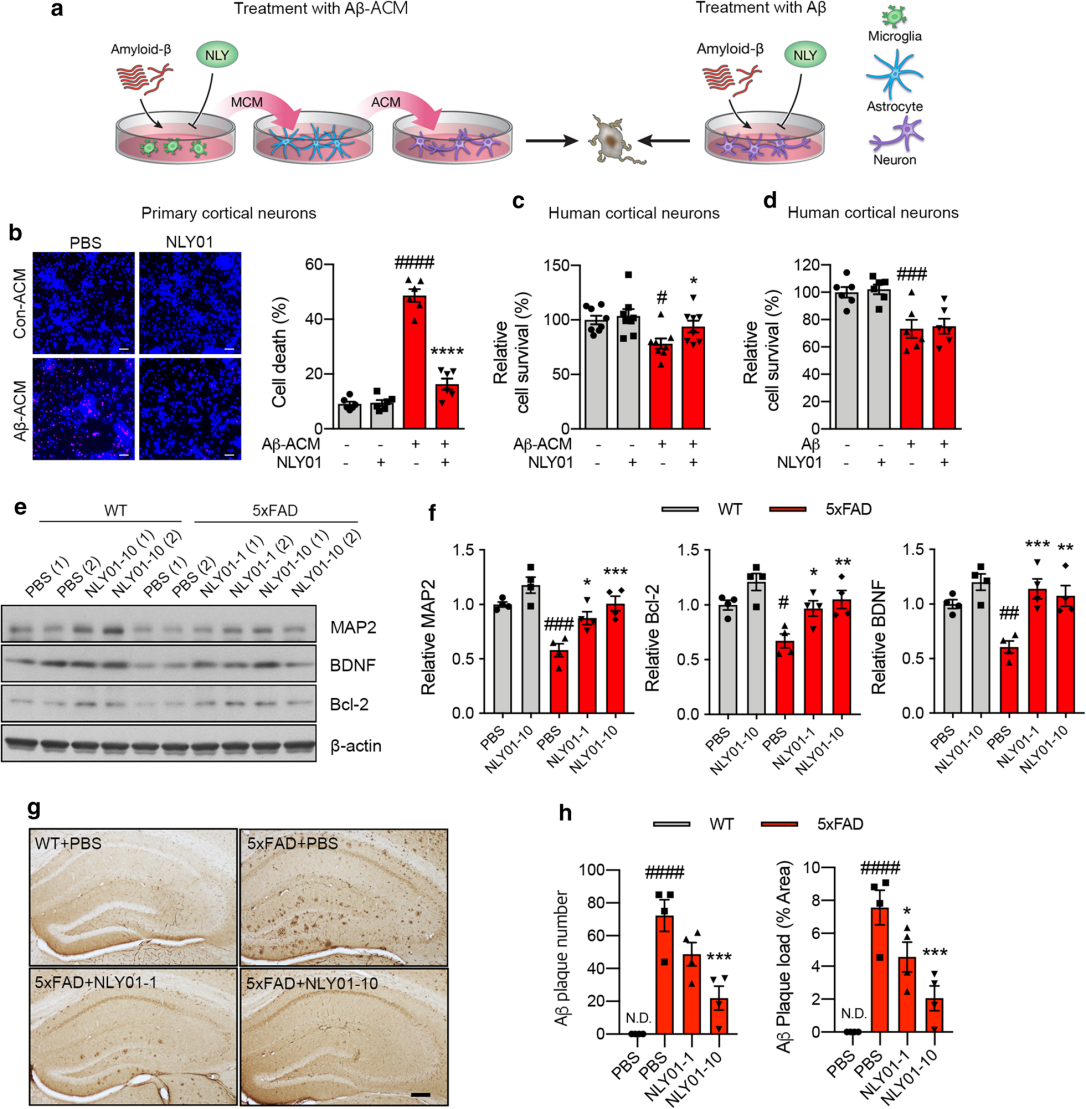
Effects of NLY01 on neuronal cell death caused by Aβ_1-42_-induced DAA. **a** Schematic diagram showing treatment of neurons with Aβ_1-42_-astrocyte conditioned media (ACM) or directly with Aβ_1-42_. **b** Representative images showing the death of mouse primary cortical neurons (left; Propidium iodide stain in red indicates the dead cells) and quantification of cell death caused by Aβ_1-42_-ACM (15 μg/ml) with or without NLY01 (1 μM) (*n* = 6, 2 technical repeats from 3 biologically independent cell cultures). **c** Quantification of cell death caused by Aβ_1-42_-ACM (50 μg/mL) with or without NLY01 (1 μM) in human cortical neurons (*n* = 8, 2 technical repeats from 4 biologically independent cell cultures). **d** Quantification of cell death caused by Aβ_1-42_ treatment (5 μM) with or without NLY01 (1 μM) in human cortical neurons (*n* = 6). **e** Protein expression of MAP2, BDNF, Bcl-2, and β-actin in the hippocampus of WT and 5xFAD mice treated with PBS or NLY01. **f** MAP2, BDNF and Bcl-2 protein levels were normalized versus β-actin levels (*n* = 4 per group). **g**, **h** Aβ plaques were stained using 4G8 antibody in WT or 5xFAD mice with NLY01 treatment. **g** Representative images of immunostaining with 4G8 (scale bar, 200 μm) and **h** quantification of Aβ plaque number and Aβ load (*n* = 4 per group). Data are shown as the mean $\pm$ SEM. *p* values were determined by one-way ANOVA. ^#^*p* < 0.05, ^##^*p* < 0.01, ^###^*p* < 0.001, ^####^*p* < 0.0001 versus Control or WT + PBS; *p < 0.05, **p < 0.01, ***p < 0.001, ****p < 0.001 versus Aβ-ACM, Aβ, or 5xFAD + PBS

Next, we investigated if prevention of reactive astrocyte conversion, induced by Aβ_1–42_ associated microglia, is neuroprotective. As shown in Fig. 5a, MCM from microglia exposed to Aβ_1–42_ in the presence or absence of NLY01 was applied to astrocytes for 24 h, followed by the collection and concentration of the astrocyte conditioned media (ACM). The concentrated ACM was then applied to primary mouse cortical cultures and human cortical neurons. Human embryonic stem cell (hESC)-derived human cortical neurons were confirmed by selective cortical markers assessed through immunocytochemical analysis (Additional file 1: Fig. S6, online resource). In a separate study, oligomeric Aβ_1–42_ was directly administered to human cortical neurons for 24 h with or without NLY01. The ACM obtained from astrocytes incubated with Aβ_1–42_ MCM (Aβ-ACM) induced neuronal cell death in mouse cortical cultures and human cortical neurons as examined by propidium iodide staining or Alamar Blue cell viability assays, respectively (Fig. 5b, c). In contrast, the ACM obtained from Aβ_1–42_ MCM incubated with NLY01 strongly reduced Aβ-ACM induced neuronal cell death (Fig. 5b, c). We also observed Aβ-ACM-induced neurite degeneration was prevented by NLY01 treatment (Additional file 1: Fig. S7a, b, online resource). Direct treatment of NLY01 in human cortical neurons incubated with oligomeric Aβ_1–42_ failed to rescue cells as assessed by the Alamar Blue assay (Fig. 5d). These results suggest that NLY01 improves neuronal viability by preventing the formation of reactive astrocytes by blocking microglia activation with limited direct effects on neurons. Consistent with the neuroprotective effects of NLY01, protein levels of MAP2 and postsynaptic density protein 95 (PSD95) were increased in 5xFAD mice treated with NLY01 (Fig. 5e, f; Additional file 1: Fig. S7c, online resource). Interestingly, NLY01 treatment also increased protein levels of brain-derived neurotrophic factor (BDNF), a key neurotrophic factor in the brain, and B-cell lymphoma 2 (Bcl-2), an anti-apoptotic protein that promotes neuron survival, in the hippocampus of both WT and 5xFAD mice (Fig. 5e, f). NLY01 treatment reduced Aβ plaque number and load in 5xFAD mice (Fig. 5g, h) as confirmed by immunohistochemistry. To further explain a reduction in plaque burden by NLY01, we performed Western blot analysis for amyloid precursor protein (APP) processing-related proteins and Aβ-degrading enzyme. As shown in Additional file 1: Fig. S8 (online resource), NLY01 treatment reduced BACE1 protein expression and C99/APP ratio as well as increased protein level of insulin degrading enzyme (IDE) in the hippocampus of 5xFAD mice.

### NLY01 rescues AD-related pathology in 3xTg-AD mice

**Fig. 6:**
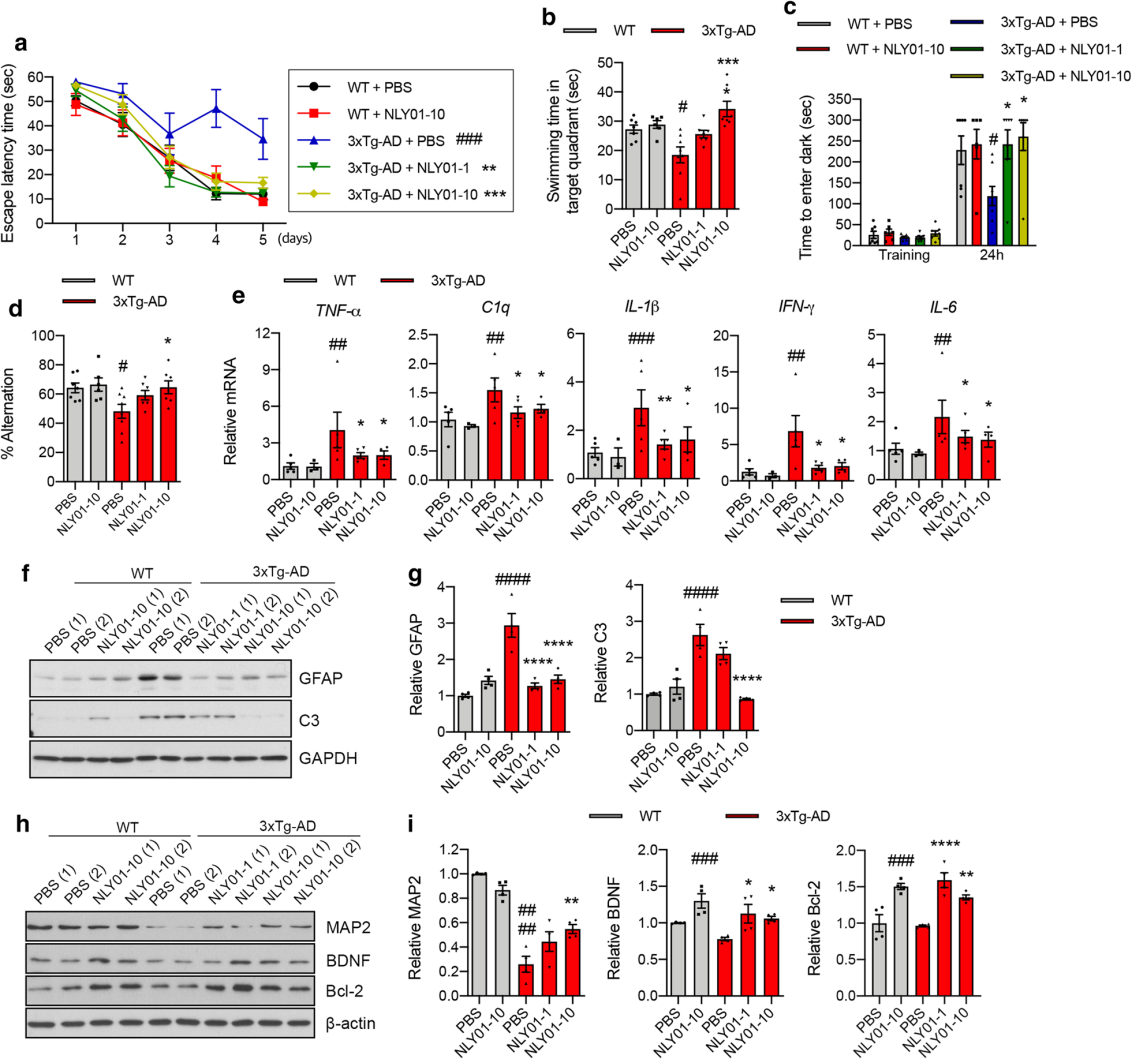
Effects of NLY01 on 3 × Tg-AD mice. 7-month-old 3 × Tg-AD mice treated with PBS or NLY01 (1 or 10 mg/kg) subcutaneously (s.c.) for 5 months (*n* = 6–8 per group). **a** Mice were trained and tested on the spatial memory version of the Morris water maze (MWM; indicated by solid lines at 60 s escape latency) (*n* = 6–8 per group). **b** Mice were given a memory probe with the platform removed at 24 h after the last training trial. 3 × Tg-AD mice treated with NLY01 exhibited dose-dependently increased time in the target quadrant (*n* = 6–8 per group). **c** Mice were placed in a light compartment and received a mild foot shock upon crossing over to the dark compartment. Mice were tested for retention of memory at 24 h after training (*n* = 6–8 per group). **d** Mice were tested on a spatial alternation task in a Y-maze (*n* = 6–8 per group). **e** The levels of *TNF-α*, *C1q*, *IL-1β*, *IFN-γ*, and *IL-6* were analyzed by qPCR in the hippocampus of mice treated with PBS or NLY01 (*n* = 3–5 per group). **f** Protein expression of GFAP and C3 in the hippocampus from WT and 3xTg-AD mice treated with PBS or NLY01 using Western blot. **g** GFAP and C3 protein levels were normalized versus GAPDH levels (*n* = 4 per group). **h** Representative images of western blot with MAP2, BDNF, and Bcl-2. **i** Protein levels of (**h**) were normalized versus β-actin levels (*n* = 4 per group). Data are shown as the mean $\pm$ SEM. *p* values were determined by one-way ANOVA. ^#^*p* < 0.05, ^##^*p* < 0.01, ^###^*p* < 0.001, ^####^*p* < 0.0001 versus WT + PBS; *p < 0.05, **p < 0.01, ***p < 0.001, ****p < 0.0001 versus 3xTg-AD + PBS

The effects of NLY01 were further investigated in 3xTg-AD mice. These mice express three mutations associated with familial AD (APP_swe_, Tau_P301L_, and Psen1_M146V_) and demonstrate learning and memory deficits at approximately 6 months of age [30]. To investigate if NLY01 can rescue behavioral deficits in the 3xTg-AD mice, treatment was initiated at 7 months of age with mice treated with either PBS or NLY01 (1 or 10 mg/kg) via s.c. injections, twice weekly, for a period of 5 months. After the treatment, behavior was evaluated in WT and 3xTg-AD mice using three different tests: MWM, passive inhibitory avoidance, and Y-maze. In the MWM, NLY01 treated 3xTg-AD mice showed a similar escape latency time compared to WT group (Fig. 6a; Additional file 1: Fig. S8a, online resource). Probe trials conducted 24 h after the last training trial demonstrated that NLY01 treatment significantly increased time in the target quadrant compared to that of PBS-treated 3xTg-AD mice (Fig. 6b; Additional file 1: Fig. S8b, online resource). No difference in swimming speed was observed (Additional file 1: Fig. S8c, online resource). In the passive inhibitory avoidance task, WT mice effectively avoided the dark, shock-associated compartment, but PBS treated 3xTg-AD mice showed impaired memory function (Fig. 6c). In contrast, NLY01 treated 3xTg-AD mice behaved in a similar manner to WT mice avoiding the dark compartment (Fig. 6c). We also observed a dose-dependent increase in Y-maze spontaneous alternation in NLY01 treated 3xTg-AD mice as compared to PBS treated 3xTg-AD mice (Fig. 6d). All mice were sacrificed after the behavioral studies. NLY01 significantly reduced mRNA expression of *TNF-α*, *C1q*, *IL-1β, IFN-γ,* and *IL-6* in the hippocampus of 3xTg-AD mice (Fig. 6e). 3xTg-AD mice showed strong GFAP and C3 inductions in the hippocampus compared to WT mice, which was blocked by NLY01 treatment (Fig. 6f, g). GFAP^+^ immunoreactivity in 3xTg-AD mice and WT was further confirmed by immunofluorescence (Additional file 1: Fig. S8d, online resource). Protein levels of MAP2, BDNF, and Bcl-2 were reduced in 3xTg-AD mice compared to WT groups and that reduction was prevented by NLY01 treatment (Fig. 6h, i). The concentrations of NLY01 in the hippocampus of 3xTg-AD mice were analyzed using exendin-4 EIA kits, resulting in the observation that NLY01 was in the brains of both WT and 3xTg-AD mice (Additional file 1: Fig. S8e, online resource). These data are consistent with previous findings that NLY01 is a CNS penetrant [38] and it prevents cognitive impairment through its pharmacological effects in the brain.

## Discussion

Microglia and astrocytes modulate neuroinflammation and neurodegeneration in the CNS. Emerging evidence on the chronic inflammatory responses and neurotoxic activities associated with the contribution of activated microglia and reactive astrocytes in the progression of disease supports the critical role of neuroinflammation in AD. During disease progression, resident microglia transform into proliferative and proinflammatory microglia with increased capacity to induce astrocyte reactivity, comprised mainly of reactive astrocytes. These activated glial cells are potential therapeutic targets for neurodegenerative disorders, including AD. Here, we show that selective blockade of Aβ-induced activation of GLP-1R^+^ microglia by subcutaneous administration of NLY01, a long-acting GLP-1R agonist, prevents reactive astrocytes conversion, neurodegeneration, and cognitive deficits without toxicity in animal models of AD.

In this study, we found that death of neurons in AD is, in part, dependent upon reactive astrocytes through Aβ-activated microglia. Targeting GLP-1R expressed on microglia by NLY01 selectively inhibited microglial activation and induction of the reactive astrocyte inducers TNF-α, C1q, and IL-1α, thus protecting both mouse and human neurons in vitro. A study with GLP-1R KO microglia further indicated the inhibitory effect of NLY01 on microglia activation is primarily through GLP-1R. Although we have used an excessive concentration of soluble Aβ oligomers, that may have limited relevance to the human AD brain, this proof-of-concept study demonstrating the effects of NLY01 in microglia and astrocytes in vitro is pertinent. Our hypothesis of targeting GLP-1R^+^ microglia by NLY01 as a potential therapeutic approach for AD was investigated in two well-established mouse models of AD, 5xFAD and 3xTg-AD mice. Both lines of mice demonstrated increased mRNA *TNF-α*, *C1q*, *IL-1α*, and *IL-1β* in the hippocampus which was significantly inhibited by NLY01 treatment. In addition, astrocytes isolated from the hippocampus of 5xFAD mice demonstrated that NLY01 treatment prevented induction of reactive astrocytes. Consequently, systemically administered NLY01 spared neurons and ameliorated behavior deficits and memory deficits in both AD mouse models. Our data indicate that the site of action of NLY01 is predominantly at glial cells and not neurons. However, how GLP-1R agonists affect or modulate the different pathways involved in glial activation needs to be studied further to fully understand the mechanisms of how NLY01 ameliorates neurodegeneration. The current study focused on the effect of the GLP-1R agonist, NLY01, on reactive astrocyte conversion by oligomeric Aβ-induced activated microglia, however, there are multiple subtypes of activated microglia and reactive astrocytes can be induced by different insults [2, 23]. Oligodendrocytes are one of the major glial cells and Aβ-induced oligodendrocyte dysfunction is strongly implicated in AD [8]. We found that GLP-1R was not expressed in oligodendrocytes of the AD mouse model. In our previous study, NLY01 reduced the Lewy body-like pathology in PD animal models associated with α-synuclein. Interestingly, in this study NLY01 treatment reduced Aβ plaque number and load in the 5xFAD mice NLY01 treatment also reduced BACE1 and increased IDE protein levels in the hippocampus of the 5xFAD. Previous studies found that GLP-1R agonists reduce Aβ plaques and neurofibrillary tangles in an AD mouse model [6, 25, 26, 36]. Whereas, other groups reported that GLP-1R agonist has no effects in Aβ deposition [4, 13]. The two animal models utilized in this study are useful for testing drug candidates for AD, however, it does not fully address the etiology of AD. Studying the role of GLP-1R agonists in additional mouse models with different features of AD including tauopathies and how ameliorating the microglia and reactive astrocytes phenotype lead to reduce Aβ deposition is warranted in future investigations.

Short-acting GLP-1R agonists, such as exendin-4 and liraglutide, have been studied in AD models and demonstrated efficacy in providing a reduction in inflammation, augmentation of mitochondrial function, and neuroprotection as well as improved behavior memory function. Although GLP-1R agonists showed promise in preclinical models, the effect of exendin-4 and liraglutide in some transgenic AD animal models was controversial [4, 13] and, importantly, the mechanisms of how GLP-1R agonists elicited therapeutic actions in the brain were not clearly reported. Our study reveals, in part, how GLP-1R agonists preserve neurons in AD and the ability of a long-acting GLP-1R agonist like NLY01 to ameliorate, in a reproducible fashion, AD pathology in two preclinical models by targeting neuroinflammation associated with microglial activation and reactive astrocytes.

## Supplementary Information

**Additional file 1.**
