# Supplementary material for: Blocking microglial activation of reactive astrocytes is neuroprotective in models of Alzheimer’s disease
Source: Acta Neuropathol Commun. 2021 Apr 26;9:78. doi: 10.1186/s40478-021-01180-z (PMC8074239; doi:10.1186/s40478-021-01180-z)
Supplement: Supplementary file 1 — Additional file 1. [file 40478_2021_1180_MOESM1_ESM.docx]

**Supplementary Information**


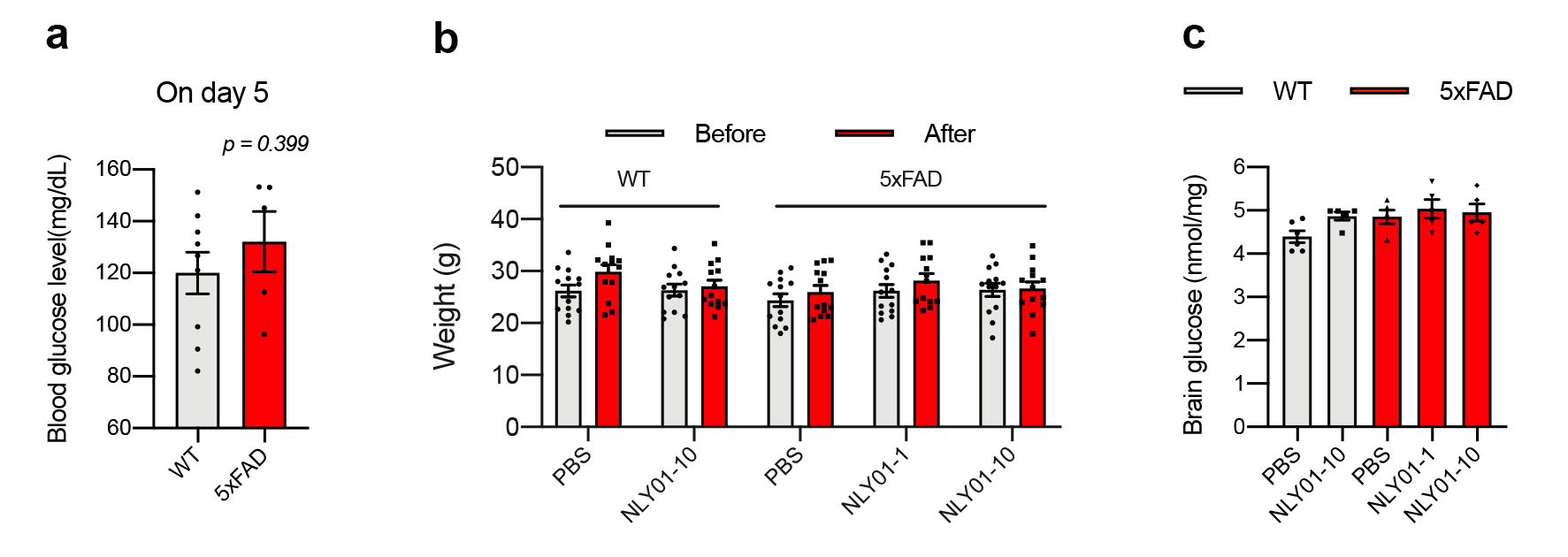


**Fig. S1** **a** The blood glucose levels in 3-month-old 5xFAD mice treated with 2 mg/kg NLY01 by intravenous injection daily for 5 days. After the last injection, blood glucose level was measured in WT and 5xFAD mice (*n* = 5-9 per group). **b, c** (**b**) Body weight changes (*n* = 13 per group) and (**c**) brain glucose levels (*n* = 5-6 per group) in the NLY01 treated mice for 4 months. Data are shown as the mean $\pm$ SEM. *p* values were determined by two-tailed unpaired t-test or one-way ANOVA.


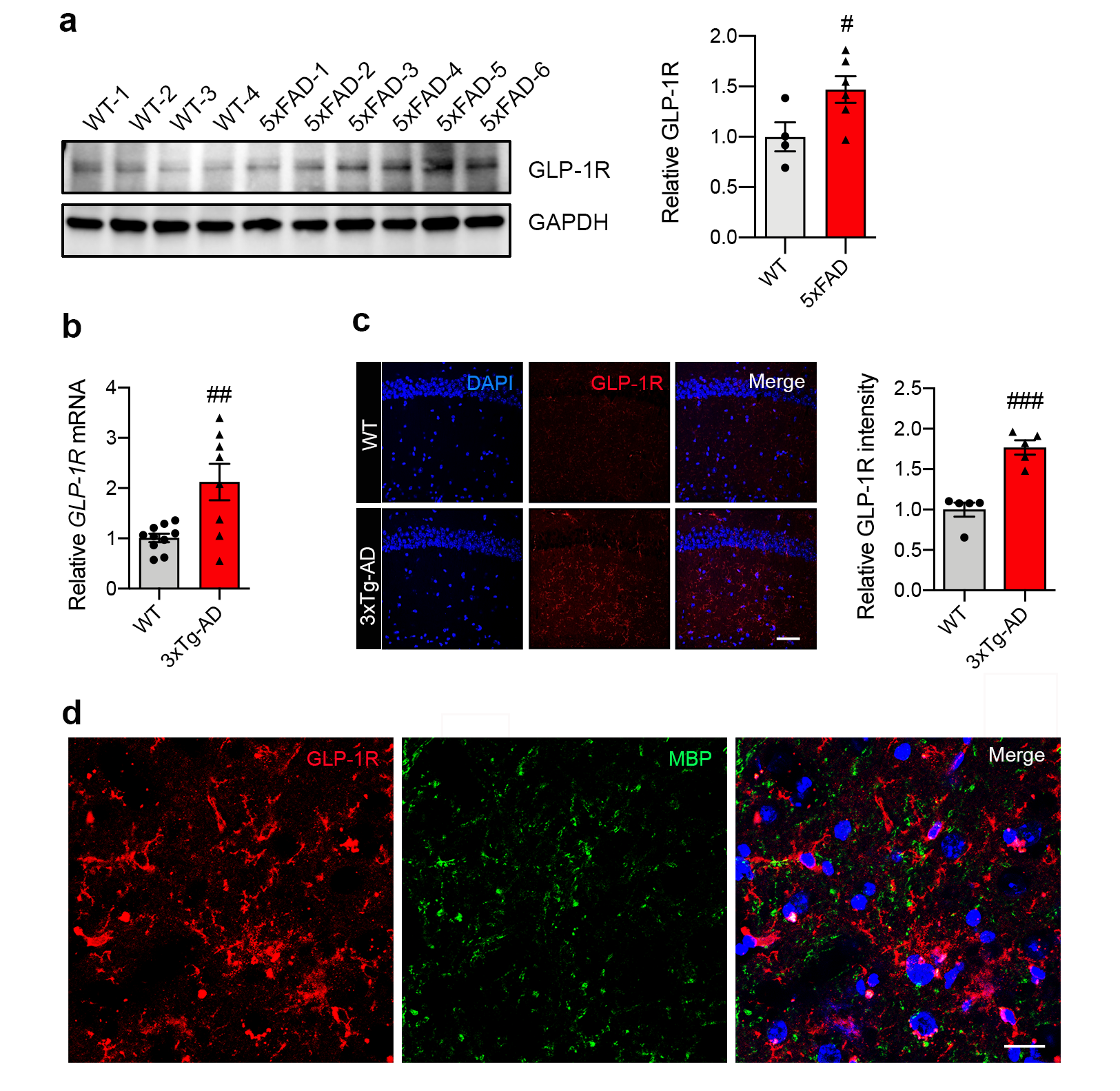


**Fig. S2** Expression of GLP-1 receptor in AD mouse models. **a** Protein level of GLP-1R in the hippocampus from the brain of 5xFAD mice. Quantification of GLP-1R is shown as relative protein expression normalized to GAPDH (7-month-old; *n* = 4-6 per group). **b** Relative *GLP-1R* mRNA expression in the hippocampus from the brain of 3xTg-AD mice (12-month-old; *n* = 8-10 per group). **c** Representative confocal images with GLP1-R (red) and DAPI (blue) in the hippocampus of 3xTg-AD mice (scale bars, 50 μm) and quantification of the GLP-1R immunostaining (*n* = 5 per group). **d** Representative images with GLP-1R (red), MBP (green), and DAPI (blue) in the hippocampus from 5xFAD mice (7-month-old; *n* = 4) (scale bar, 20 μm). Data are shown as the mean $\pm$ SEM. *p* values were determined by two-tailed unpaired t-test. ^#^*p* < 0.05, ^##^*p* < 0.01, ^###^*p* < 0.001 versus WT


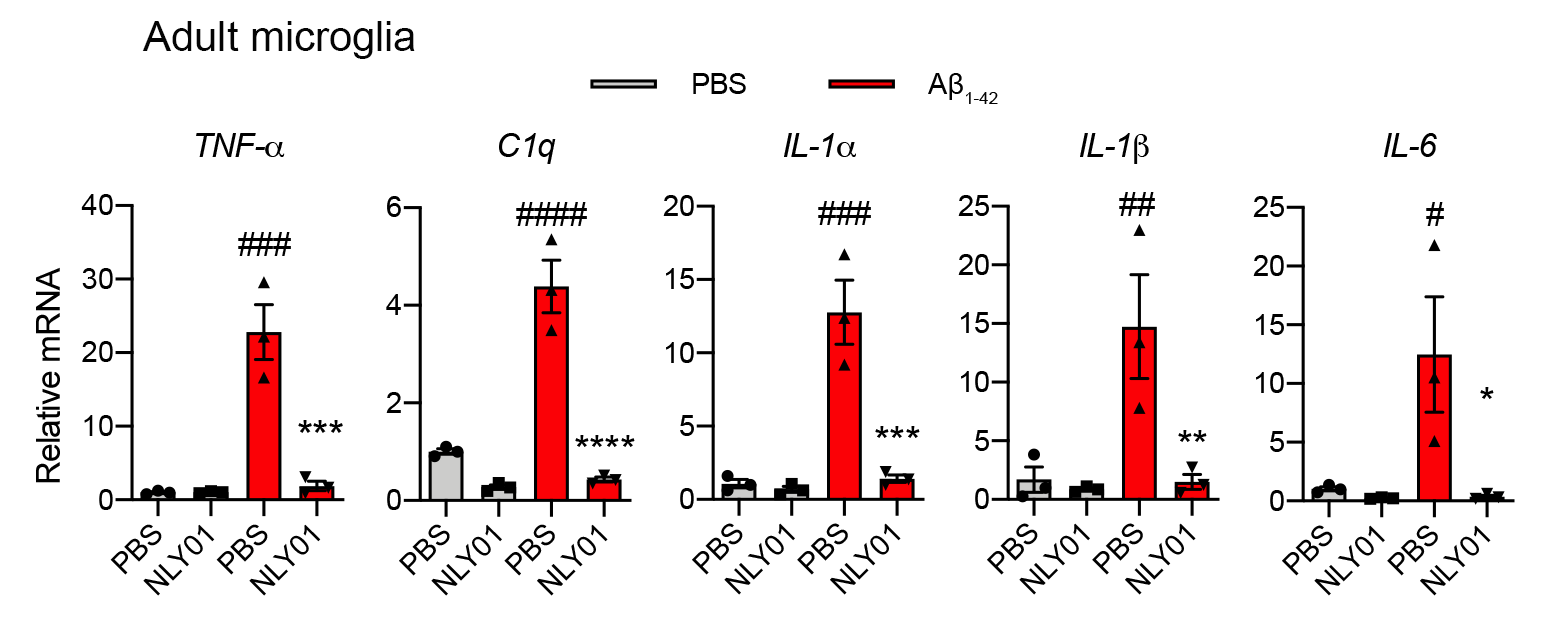


**Fig. S3** NLY01 suppresses Aβ-induced adult microglia activation. Adult primary microglia were pre-treated with PBS or NLY01 (1 μM) for 30 minutes, and then further incubated with oligomeric Aβ_1-42_ (1 μM) for 4 hours. mRNAs levels of *TNF-α*, *C1q*, *IL-1α*, *IL-1β*, and *IL-6* were determined using qPCR (*n* = 3 biologically independent cell cultures). Data are shown as the mean $\pm$ SEM. *p* values were determined by one-way ANOVA. ^#^*p* < 0.05, ^##^*p* < 0.01, ^###^*p* < 0.001, ^####^*p* < 0.0001 versus Control; ^*^*p* < 0.05, ^**^*p* < 0.01, ^***^*p* < 0.001, ^****^*p* < 0.0001 versus Aβ_1-42_ + PBS.


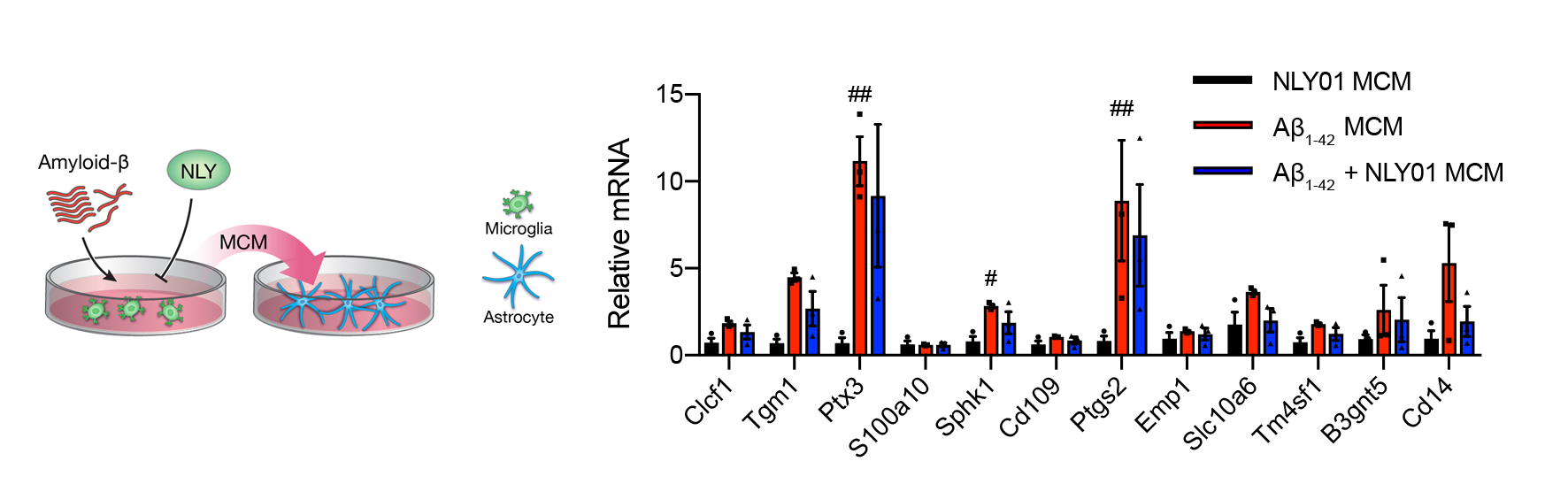


**Fig. S4** Schematic diagram showing the treatment of astrocytes with MCM from Aβ_1-42_ treated microglia with or without NLY01. mRNA levels of primary astrocytes 24 hours post treatment with MCM from oligomeric Aβ_1-42_ (1 μM)-activated microglia with or without NLY01 (1 μM) are shown (*n* = 3 biologically independent cell cultures). Data are shown as the mean $\pm$ SEM. *p* values were determined by one-way ANOVA. ^#^*p* < 0.05, ^##^*p* < 0.01 versus Control.


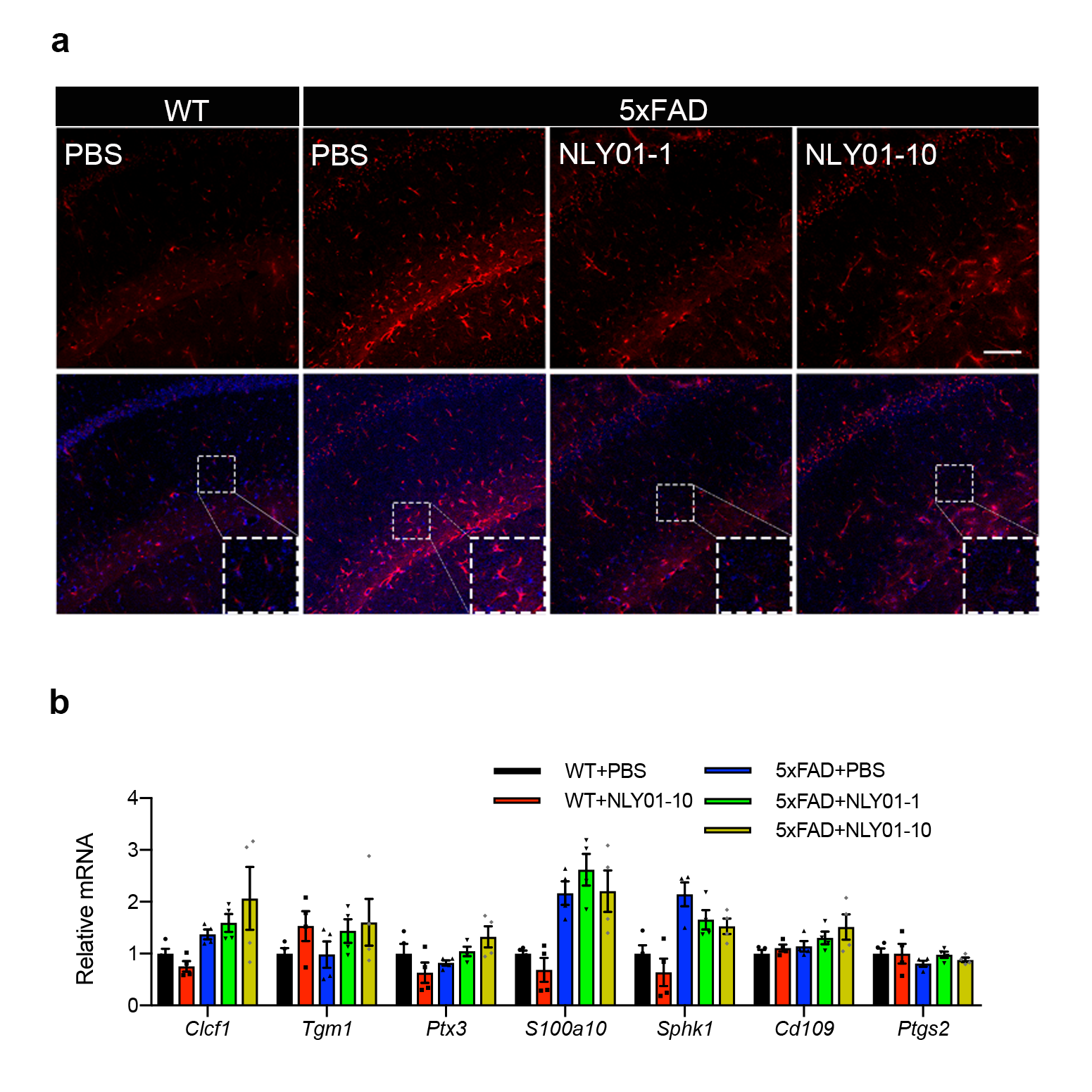


**Fig. S5** Reactive astrocyte changes in 5xFAD mice by NLY01. 3-month-old 5xFAD mice were treated with PBS or NLY01 (1 or 10 mg/kg) subcutaneously (s.c.) for 4 months (*n* = 9-13 per group). **a** Representative images of immunostaining with GFAP (red) and DAPI (blue) in the hippocampus of WT and 5xFAD mice treated with NLY01 (scale bar, 100 μm). **b** mRNA levels of astrocyte signatures in the hippocampus of WT and 5xFAD mice treated with PBS or NLY01 were measured using qPCR. GAPDH was used to normalize for the amounts of cDNA (*n* = 4 per group). Data are shown as the mean $\pm$ SEM. *p* values were determined by one-way ANOVA.

**
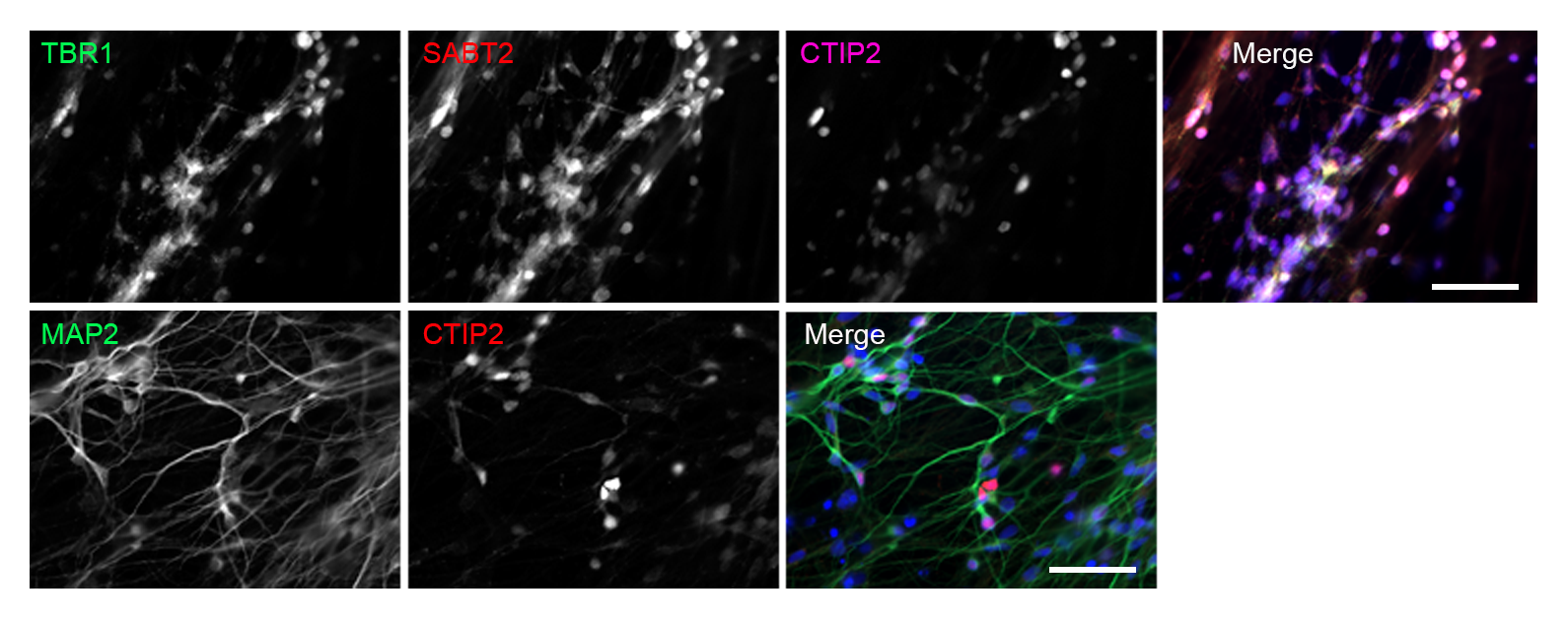
**

**Fig. S6** Immunocytochemical analysis of mature neuronal markers in human cortical neurons (scale bar, 50 μm).


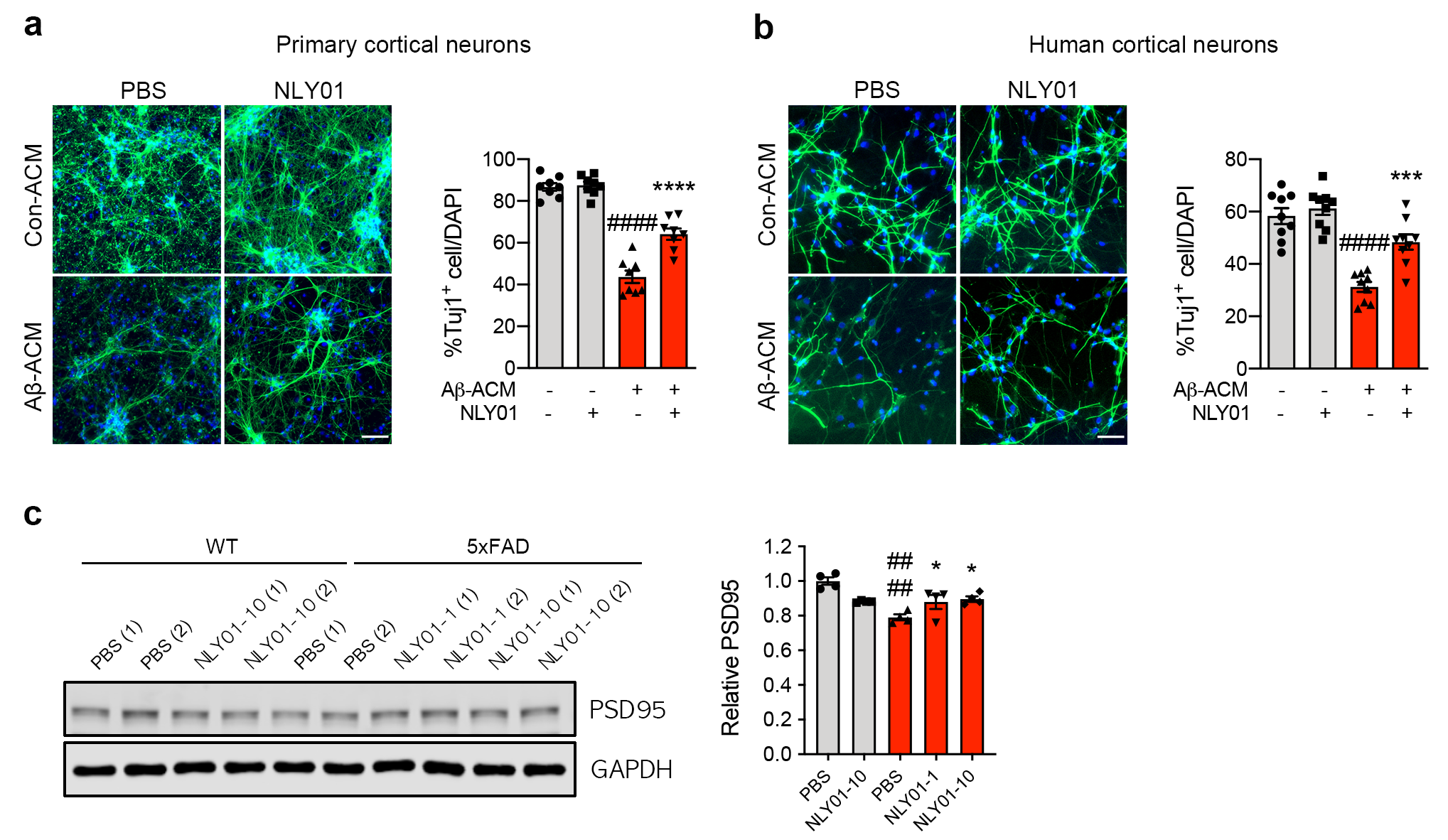


**Fig. S7** NLY01 protects neuron and synaptic loss. **a, b** Immunostaining of Tuj1, neuronal marker, in (**a**) mouse primary cultured neurons and (**b**) human cortical neurons (scale bar, 50 μm). Tuj1^+^ cells were quantified (*n* = 8-9, 2 technical repeats from 4 biologically independent cell cultures). **c** Protein expression of PSD95 and GAPDH in the hippocampus of WT and 5xFAD mice treated with PBS or NLY01. PSD95 protein levels were normalized versus GAPDH levels (*n* = 4 per group). Data are shown as the mean $\pm$ SEM. *p* values were determined by one-way ANOVA. ^####^*p* < 0.0001 versus WT+PBS; ^*^*p* < 0.05 versus 5xFAD + PBS.


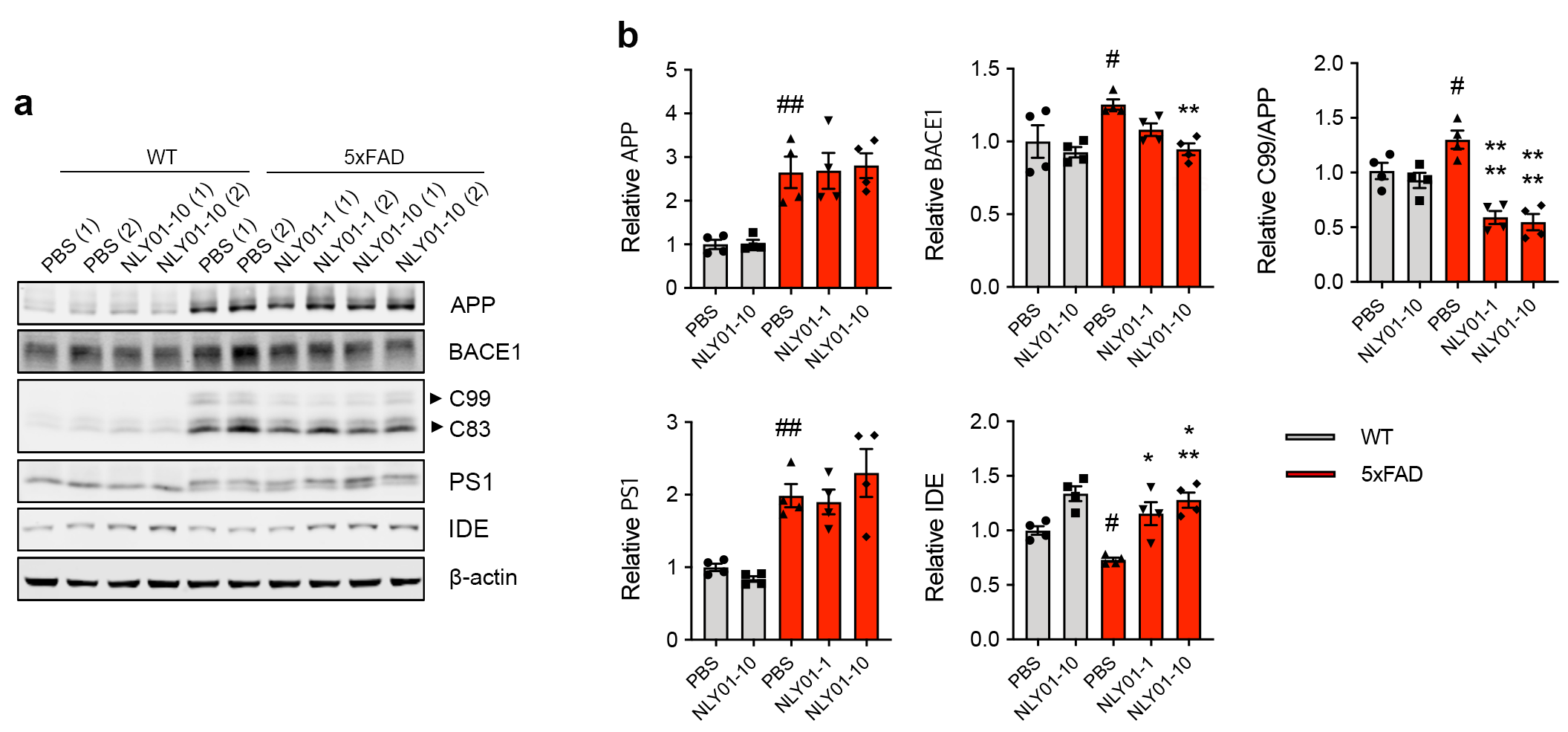


**Fig. S8** **a** Protein expression of APP, BACE1, APP-CTFs (C99 and C83), PS1, IDE and β-actin in the hippocampus of WT and 5xFAD mice treated with PBS or NLY01. **b** All protein levels were normalized versus β-actin levels (n = 4 per group). Data are shown as the mean $\pm$ SEM. *p* values were determined by one-way ANOVA. ^#^*p* < 0.05, ^##^*p* < 0.01 versus WT+PBS; *p* < 0.05, ^**^*p* < 0.01, ^***^*p* < 0.001, ^****^*p* < 0.0001 versus 5xFAD + PBS.


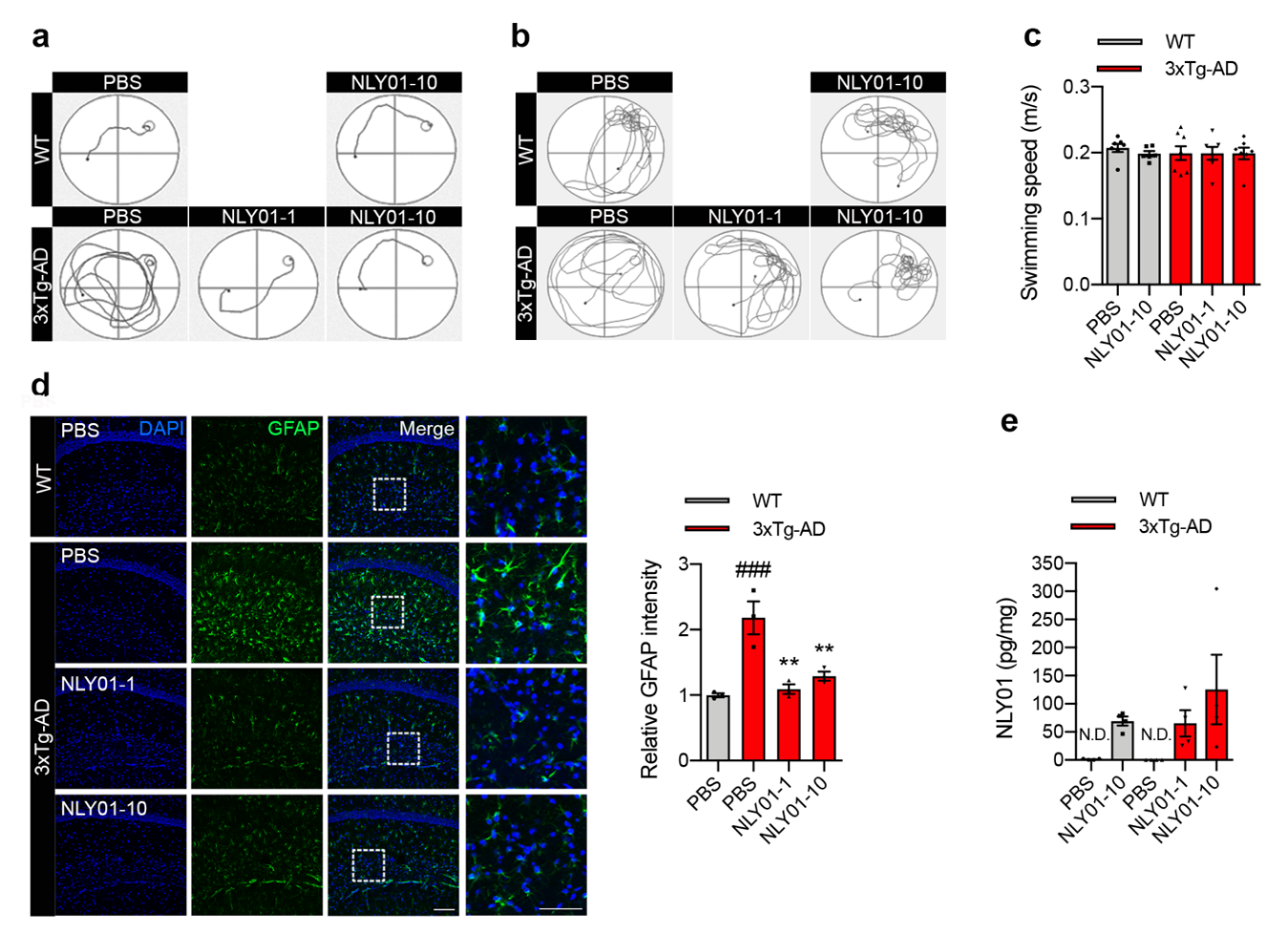


**Fig. S9** Effects of NLY01 in 3xTg-AD mice. 7-month-old 3xTg-AD mice treated with PBS or NLY01 (1 or 10 mg/kg) subcutaneously (s.c.) for 5 months (*n* = 6-8 per group). **a-c** Mice were trained and tested on the spatial memory version of the Morris water maze (MWM) (*n* = 6-8 per group). (**a**) Representative swimming traces for training trials on day 5 are shown. (**b)** Representative swimming traces at 24 hours after the last training trial. (**c**) No difference in swimming speed was observed. **d** Representative images of immunostaining with GFAP (green) and DAPI (blue) in the hippocampus of WT and 3xTg-AD mice treated with NLY01 (scale bar, left: 50 μm; right: 100 μm). **e** NLY01 accumulation was quantified in the hippocampus from these mice using an Exendin-4 EIA kit (*n* = 4 per group). Data are shown as the mean $\pm$ SEM. *p* values were determined by one-way ANOVA. ^###^*p* < 0.001 versus WT + PBS; ^**^*p* < 0.01 versus 3xTg-AD + PBS.

**Table. S1** Detailed patient information

| **Number** | **Samples** | **Age** | **Sex** | **Race** | **PMD** | **FRZ** | **CERAD score** | **Braak**  **stage** |
| --- | --- | --- | --- | --- | --- | --- | --- | --- |
| 4 | Control | 63 | F | W | 22 | Hippocampus | 0 | I |
| 5 | Control | 69 | F | W | 13 | Hippocampus | 0 | 0 |
| 10 | Control | 67 | M | B | 23 | Hippocampus | 0 | I |
| 12 | Control | 64 | M | W | 11 | Hippocampus | 0 | I |
| 13 | Control | 59 | M | B | 20 | Hippocampus | 0 | I |
| 40 | Control | 72 | N/A | N/A | N/A | Hippocampus | 0 | I |
| 2282 | AD | 79 | F | W | 18 | Hippocampus | C | VI |
| 2417 | AD | 61 | F | W | 5 | Hippocampus | B | VI |
| 2430 | AD | 62 | M | N/A | 13 | Hippocampus | C | VI |
| 2447 | AD | 65 | M | W | 11 | Hippocampus | C | VI |
| 2454 | AD | 62 | F | W | 18 | Hippocampus | C | VI |
| 2464 | AD | 52 | M | W | 5 | Hippocampus | C | VI |

**Table. S2** Sequence of PCR primers used in qPCR.

| **Genes** | **Forward** | **Reverse** |
| --- | --- | --- |
| **Human** | | |
| GLP-1R | TTGGGGTGAACTTCCTCATC | CTTGGCAAGTCTGCATTTGA |
| 18sRNA | CTACCACATCCAAGGAAGCA | TTTTTCGTCACTACCTCCCCG |
| **Mouse** | | |
| GLP-1R | GGGTCTCTGGCTACATAAGGAC | AAGGATGGCTGAAGCGATGAC |
| TNF-α | TCTCATGCACCACCATCAAGGACT | ACCACTCTCCCTTTGCAGAACTCA |
| C1q | CTCAGGGATGGCTGGTGGCC | CCTTTGAGACCCGGCCTCCCC |
| IL-1β | CAACCAACAAGTGATATTCTCCATG | GATCCACACTCTCCAGCTGCA |
| IFN-γ | CAGCAACAGCAAGGCGAAA | CTGGACCTGTGGGTTGTTGAC |
| IL-6 | GGTGACAACCACGGCCTTCCC | TTAAGCCTCCGACTTGTGAAGTGGT |
| IL-1α | CGGGTGACAGTATCAGCAAC | GACAAACTTCTGCCTGACGA |
| Lcn2 | CCAGTTCGCCATGGTATTTT | CACACTCACCACCCATTCAG |
| Steap4 | CCCGAATCGTGTCTTTCCTA | GGCCTGAGTAATGGTTGCAT |
| S1pr3 | AAGCCTAGCGGGAGAGAAAC | TCAGGGAACAATTGGGAGAG |
| Timp1 | AGTGATTTCCCCGCCAACTC | GGGGCCATCATGGTATCTGC |
| Hspb1 | GACATGAGCAGTCGGATTGA | GGATGGGGTGTAGGGGTACT |
| Cxcl10 | CCCACGTGTTGAGATCATTG | CACTGGGTAAAGGGGAGTGA |
| Cd44 | ACCTTGGCCACCACTCCTAA | GCAGTAGGCTGAAGGGTTGT |
| Osmr | GTGAAGGACCCAAAGCATGT | GCCTAATACCTGGTGCGTGT |
| Cp | TGTGATGGGAATGGGCAATGA | AGTGTATAGAGGATGTTCCAGGTCA |
| Serpina3n | CCTGGAGGATGTCCTTTCAA | TTATCAGGAAAGGCCGATTG |
| Aspg | GCTGCTGGCCATTTACACTG | GTGGGCCTGTGCATACTCTT |
| Vim | AGACCAGAGATGGACAGGTGA | TTGCGCTCCTGAAAAACTGC |
| Gfap | AGAAAGGTTGAATCGCTGGA | CGGCGATAGTCGTTAGCTTC |
| H2-T.23 | GGACCGCGAATGACATAGC | GCACCTCAGGGTGACTTCAT |
| Serping1 | ACAGCCCCCTCTGAATTCTT | GGATGCTCTCCAAGTTGCTC |
| H2-D1 | TCCGAGATTGTAAAGCGTGAAGA | ACAGGGCAGTGCAGGGATAG |
| Ggta1 | GTGAACAGCATGAGGGGTTT | GTTTTGTTGCCTCTGGGTGT |
| Ligp1 | GGGGCAATAGCTCATTGGTA | ACCTCGAAGACATCCCCTTT |
| Gbp2 | GGGGTCACTGTCTGACCACT | GGGAAACCTGGGATGAGATT |
| Fbln5 | CTTCAGATGCAAGCAACAA | AGGCAGTGTCAGAGGCCTTA |
| Ugt1a | CCTATGGGTCACTTGCCACT | AAAACCATGTTGGGCATGAT |
| Fkbp5 | TATGCTTATGGCTCGGCTGG | CAGCCTTCCAGGTGGACTTT |
| Psmb8 | CAGTCCTGAAGAGGCCTACG | CACTTTCACCCAACCGTCTT |
| Srgn | GCAAGGTTATCCTGCTCGGA | TGGGAGGGCCGATGTTATTG |
| Amigo2 | GAGGCGACCATAATGTCGTT | GCATCCAACAGTCCGATTCT |
| C3 | CCAGCTCCCCATTAGCTCTG | GCACTTGCCTCTTTAGGAAGTC |
| Clcf1 | CTTCAATCCTCCTCGACTGG | TACGTCGGAGTTCAGCTGTG |
| Tgm1 | CTGTTGGTCCCGTCCCAAA | GGACCTTCCATTGTGCCTGG |
| Ptx3 | AACAAGCTCTGTTGCCCATT | TCCCAAATGGAACATTGGAT |
| S100a10 | CCTCTGGCTGTGGACAAAAT | CTGCTCACAAGAAGCAGTGG |
| Sphk1 | GATGCATGAGGTGGTGAATG | TGCTCGTACCCAGCATAGTG |
| Cd109 | CACAGTCGGGAGCCCTAAAG | GCAGCGATTTCGATGTCCAC |
| Ptgs2 | GCTGTACAAGCAGTGGCAAA | CCCCAAAGATAGCATCTGGA |
| Emp1 | GAGACACTGGCCAGAAAAGC | TAAAAGGCAAGGGAATGCAC |
| Slc10a6 | GCTTCGGTGGTATGATGCTT | CCACAGGCTTTTCTGGTGAT |
| Tm4sf1 | GCCCAAGCATATTGTGGAGT | AGGGTAGGATGTGGCACAAG |
| B3gnt5 | CGTGGGGCAATGAGAACTAT | CCCAGCTGAACTGAAGAAGG |
| Cd14 | GGACTGATCTCAGCCCTCTG | GCTTCAGCCCAGTGAAAGAC |
| GAPDH | TTGATGGCAACAATCTCCAC | CGTCCCGTAGACAAAATGGT |
| 18sRNA | GTAACCCGTTGAACCCCATT | CCATCCAATCGGTAGTAGCG |

**Table. S3** Antibodies used in this study.

| **Antibodies** | **Source/Cat. No.** | **Host** | **Dilution** |
| --- | --- | --- | --- |
| GLP-1R | Santa Cruz (sc-390774) | Mouse | 1:1000 (WB), 1:300 (IF) |
| Iba-1 | Abcam (ab178847) | Rabbit | 1:1000 (WB), 1:200 (IF) |
| GFAP | Cell signaling (3670) | Mouse | 1:1000 (WB), 1:300 (IF) |
| MBP | Invitrogen (PA5-78397) | Rabbit | 1:200 (IF) |
| TBR1 | Abcam (ab31940) | Rabbit | 1:250 (IF) |
| CTIP2 | Abcam (ab18465) | Rat | 1:250 (IF) |
| SATB2 | Abcam (ab51502) | Mouse | 1:250 (IF) |
| MAP2 | Cell signaling (4542) | Rabbit | 1:1000 (WB), 1:50 (IF) |
| 4G8 | BioLegend (800720) | Mouse | 1:1000 (IHC) |
| Tuj1 | BioLegend (801206) | Mouse | 1:1000 (WB) |
| C3 | Abcam (ab200999) | Rabbit | 1:1000 (WB) |
| BDNF | Abcam (ab108319) | Rabbit | 1:1000 (WB) |
| Bcl-2 | Cell signaling (3498) | Rabbit | 1:1000 (WB) |
| PSD95 | Cell signaling (3450) | Rabbit | 1:1000 (WB) |
| β-actin | Santa Cruz (sc-47778) | Mouse | 1:10000 (WB) |
| GAPDH | Santa Cruz (sc-32233) | Mouse | 1:10000 (WB) |

**Table. S4** mRNA expression of astrocyte signatures in the primary astrocytes. Data are shown as the mean $\pm$ SEM. (*n* = 3). *p* values were determined by one-way ANOVA. ^#^*p* < 0.05, ^##^*p* < 0.01, ^###^*p* < 0.001, ^####^*p* < 0.0001 versus Con MCM; ^*^*p* < 0.05, ^**^*p* < 0.01, ^***^*p* < 0.001 versus Aβ_1-42_ MCM.

| **mRNA** | **NLY01 MCM** | **Aβ_1-42_ MCM** | **Aβ_1-42_+ NLY01 MCM** |
| --- | --- | --- | --- |
| *Lcn2* | 1.16 ± 0.32 | 660.08 ± 83.02^####^ | 191.26 ± 36.47^***^ |
| *Steap4* | 1.36 ± 0.44 | 39.28 ± 7.47^##^ | 20.39 ± 10.12 |
| *S1pr3* | 1.02 ± 0.15 | 11.35 ± 3.81^#^ | 2.65 ± 1.08^*^ |
| *Timp1* | 1.42 ± 0.16 | 16.67 ± 7.32 | 7.74 ± 3.64 |
| *Hspb1* | 1.13 ± 0.14 | 5.19 ± 1.77^#^ | 2.14 ± 0.77 |
| *Cxcl10* | 1.20 ± 0.36 | 47.88 ± 0.74^####^ | 16.69 ± 6.56^***^ |
| *Cd44* | 0.71 ± 0.28 | 9.29 ± 6.47 | 2.19 ± 1.24 |
| *Osmr* | 0.68 ± 0.25 | 7.15 ± 0.17^###^ | 3.11 ± 1.09^**^ |
| *Cp* | 0.62 ± 0.23 | 3.30 ± 0.79^#^ | 1.04 ± 0.30^*^ |
| *Serpina3n* | 0.68 ± 0.24 | 5.67 ± 1.02^##^ | 1.62 ± 0.49^**^ |
| *Aspg* | 0.87 ± 0.34 | 1.36 ± 0.07 | 0.88 ± 0.23 |
| *Vim* | 0.72 ± 0.25 | 1.90 ± 0.47 | 0.92 ± 0.30 |
| *Gfap* | 0.65 ± 0.23 | 0.83 ± 0.04 | 0.57 ± 0.21 |
| *H2.T23* | 1.40 ± 0.94 | 20.84 ± 5.86^##^ | 5.00 ± 0.92^*^ |
| *Serping1* | 1.00 ± 0.56 | 16.49 ± 5.83^#^ | 3.38 ± 0.88^*^ |
| *H2.D1* | 1.12 ± 0.65 | 13.62 ± 2.89^##^ | 3.39 ± 0.76^**^ |
| *Ggta1* | 1.41 ± 0.86 | 5.88 ± 0.27^###^ | 1.54 ± 0.15^***^ |
| *Ligp1* | 1.54 ± 0.82 | 34.46 ± 6.17^###^ | 6.68 ± 1.05^***^ |
| *Gbp2* | 1.01 ± 0.60 | 5.68 ± 1.61^#^ | 1.77 ± 0.29^*^ |
| *Fbln5* | 0.80 ± 0.38 | 0.79 ± 0.08 | 0.64 ± 0.11 |
| *Ugt1a* | 1.89 ± 0.88 | 1.51 ± 0.11 | 0.76 ± 0.18 |
| *Fkbp5* | 1.07 ± 0.54 | 0.91 ± 0.25 | 0.53 ± 0.12 |
| *Psmb8* | 1.40 ± 0.85 | 17.23 ± 3.69^###^ | 3.08 ± 0.56^**^ |
| *Srgn* | 1.71 ± 1.06 | 5.56 ± 1.32^#^ | 2.10 ± 0.39 |
| *Amigo2* | 0.95 ± 0.34 | 2.64 ± 0.08^##^ | 1.13 ± 0.33^**^ |
| *C3* | 0.61 ± 0.10 | 5.61 ± 0.61^####^ | 1.97 ± 0.32^****^ |
| *Clcf1* | 0.73 ± 0.26 | 1.56 ± 0.02 | 0.88 ± 0.25 |
| *Tgm1* | 0.70 ± 0.23 | 2.25 ± 0.25 | 1.38 ± 0.40 |
| *Ptx3* | 0.70 ± 0.31 | 8.82 ± 1.67^##^ | 5.13 ± 1.79 |
| *S100a10* | 0.63 ± 0.21 | 0.58 ± 0.02 | 0.45 ± 0.17 |
| *Sphk1* | 0.79 ± 0.28 | 2.02 ± 0.04^#^ | 1.20 ± 0.36 |
| *Cd109* | 0.61 ± 0.22 | 0.98 ± 0.05 | 0.58 ± 0.19 |
| *Ptgs2* | 0.81 ± 0.29 | 5.99 ± 1.08^##^ | 3.52 ± 1.19 |
| *Emp1* | 0.95 ± 0.35 | 1.23 ± 0.03 | 0.77 ± 0.22 |
| *Slc10a6* | 1.77 ± 0.71 | 2.09 ± 0.13 | 1.02 ± 0.30 |
| *Tm4sf1* | 0.75 ± 0.26 | 1.22 ± 0.02 | 0.75 ± 0.23 |
| *B3gnt5* | 0.93 ± 0.25 | 1.64 ± 0.72 | 1.22 ± 0.73 |
| *Cd14* | 0.96 ± 0.47 | 3.26 ± 1.32 | 1.26 ± 0.56 |

**Table. S5** mRNA expression of astrocyte signatures in 5xFAD mice. Data are shown as the mean $\pm$ SEM. (*n* = 4 per group). *p* values were determined by one-way ANOVA. ^##^*p* < 0.01, ^###^*p* < 0.001, ^####^*p* < 0.0001 versus WT+PBS; ^*^*p* < 0.05, ^**^*p* < 0.01, ^***^*p* < 0.001, ^****^*p* < 0.0001 versus 5xFAD+PBS.

| **mRNA** | **WT** | | **5xFAD** | | |
| --- | --- | --- | --- | --- | --- |
|  | **PBS** | **NLY01-10** | **PBS** | **NLY01-1** | **NLY01-10** |
| *Lcn2* | 1 ± 0.23 | 1.4 ± 0.35 | 43.61 ± 2.18^####^ | 13.55 ± 2.21^****^ | 12.11 ± 2.61^****^ |
| *Steap4* | 1 ± 0.22 | 0.55 ± 0.12 | 8.47 ± 1.85^####^ | 3.40 ± 0.43^**^ | 2.31 ± 0.72^***^ |
| *S1pr3* | 1 ± 0.11 | 0.81 ± 0.17 | 7.73 ± 0.95^####^ | 5.03 ± 0.91 | 2.91 ± 0.46^**^ |
| *Timp1* | 1 ± 0.26 | 0.53 ± 0.26 | 4.51 ± 0.43^####^ | 2.93 ± 0.31^*^ | 2.51 ± 0.92^*^ |
| *Hspb1* | 1 ± 0.06 | 0.96 ± 0.10 | 2.64 ± 0.16^####^ | 1.44 ± 0.16^**^ | 1.41 ± 0.30^***^ |
| *Cxcl10* | 1 ± 0.12 | 0.91 ± 0.38 | 27.75 ± 3.13^####^ | 7.40 ± 0.73^****^ | 5.49 ± 1.38^****^ |
| *Osmr* | 1 ± 0.09 | 0.73 ± 0.17 | 5.89 ± 1.48^###^ | 2.72 ± 0.47^*^ | 2.22 ± 0.50^**^ |
| *H2-T23* | 1 ± 0.37 | 1.15 ± 0.22 | 7.54 ± 1.14^####^ | 3.03 ± 0.69^**^ | 2.85 ± 0.54^**^ |
| *Serping1* | 1 ± 0.02 | 1.07 ± 0.18 | 6.37 ± 1.18^####^ | 3.52 ± 0.44^*^ | 2.12 ± 0.52^***^ |
| *H2-D1* | 1 ± 0.22 | 1.21 ± 0.40 | 4.71 ± 0.67^##^ | 2.49 ± 0.26 | 2.92 ± 1.23 |
| *Ggta1* | 1 ± 0.04 | 0.62 ± 0.12 | 5.83 ± 0.98^####^ | 4.04 ± 0.35 | 2.49 ± 1.10^**^ |
| *Ligp1* | 1 ± 0.16 | 0.96 ± 0.19 | 4.37 ± 1.55 | 2.45 ± 1.36 | 2.68 ± 1.06 |
| *Gbp2* | 1 ± 0.10 | 0.90 ± 0.18 | 5.70 ± 0.58^####^ | 3.69 ± 0.21 | 2.30 ± 0.73^**^ |
| *Fbln5* | 1 ± 0.07 | 0.77 ± 0.04 | 5.33 ± 0.70^####^ | 4.12 ± 0.60 | 3.02 ± 0.93^*^ |
| *Clcf1* | 1 ± 0.10 | 0.75 ± 0.10 | 1.37 ± 0.09 | 1.60 ± 0.17 | 2.07 ± 0.61 |
| *Tgm1* | 1 ± 0.11 | 1.53 ± 0.29 | 0.98 ± 0.26 | 1.44 ± 0.23 | 1.61 ± 0.45 |
| *Ptx3* | 1 ± 0.19 | 0.63 ± 0.20 | 0.82 ± 0.05 | 1.05 ± 0.09 | 1.33 ± 0.20 |
| *S100a10* | 1 ± 0.06 | 0.69 ± 0.23 | 2.17 ± 0.23 | 2.62 ± 0.31 | 2.21 ± 0.40 |
| *Sphk1* | 1 ± 0.16 | 0.64 ± 0.26 | 2.15 ± 0.23 | 1.65 ± 0.19 | 1.53 ± 0.15 |
| *Cd109* | 1 ± 0.07 | 1.11 ± 0.07 | 1.14 ± 0.10 | 1.30 ± 0.12 | 1.52 ± 0.24 |
| *Ptgs2* | 1 ± 0.10 | 1 ± 0.19 | 0.81 ± 0.06 | 0.98 ± 0.06 | 0.88 ± 0.05 |
